# Exploring causal links between multifaceted dietary exposures and stroke subtypes: Results from a two-sample Mendelian randomization analysis

**DOI:** 10.1097/MD.0000000000048375

**Published:** 2026-04-17

**Authors:** Zhibo Xuan, Huasen Yang, Lining Duan, Mengwan Hu, Xian Wu, Weiwei Shan

**Affiliations:** a Heilongjiang University of Chinese Medicine, Harbin, China; b First Affiliated Hospital of Guangzhou University of Chinese Medicine, Guangzhou, China; c First Affiliated Hospital of Heilongjiang University of Chinese Medicine, Harbin, China.

**Keywords:** causal relationship, dietary intake, genome-wide association studies, Mendelian randomization, stroke subtypes

## Abstract

While observational studies have identified a link between dietary factors and the incidence of stroke, whether this association reflects a causal relationship remains poorly defined. For this work, we utilized a two-sample Mendelian randomization (MR) method to investigate the potential causal association between dietary intake and the risk of stroke. Genetic instrumental variables were extracted from the IEU OpenGWAS and GWAS Catalog databases. The inverse variance weighted method was used to calculate the MR estimates, and sensitivity analyses were performed to evaluate the robustness of the results. Dried fruit intake was associated with a reduced risk of stroke (odds ratio [*OR*] = 0.991, 95% confidence interval [95% *CI*]: 0.983–0.998, *P* = .013) and its subtypes, including ischemic stroke (*OR* = 0.634, 95% *CI*: 0.417–0.965, *P* = .034), small-vessel ischemic stroke (*OR* = 0.381, 95% *CI*: 0.195–0.743, *P* = .005), and lacunar stroke (*OR* = 0.320, 95% *CI*: 0.149–0.686, *P* = .003). Oily fish intake exerted a protective effect against stroke (*OR* = 0.994, 95% *CI*: 0.988–0.999; *P* = .016). Non-oily fish intake reduced the risk of lacunar stroke (*OR* = 0.256, 95% *CI*: 0.081–0.807, *P* = .020), and pork intake reduced the risk of intracerebral hemorrhage (*OR* = 0.169, 95% *CI*: 0.031–0.930, *P* = .041). No significant causal associations were found between stroke and the intakes of processed meat, salad/raw vegetables, fresh fruits, beef, cooked vegetables, poultry, lamb/mutton, or coffee. Reverse MR analyses revealed no widespread and robust reverse causal associations; only 2 nominally significant signals were detected, which had poor analytical robustness or negligible clinical relevance and did not affect the core forward causal inferences. This study provides genetic evidence supporting the causal relationships between specific dietary factors (dried fruit, oily fish, nonoily fish, and pork) and stroke risk, which may have implications for dietary guidance and stroke prevention strategies.

## 1. Introduction

Stroke is one of the most critical health challenges globally, posing a substantial threat to human health and safety. As a major global health challenge, stroke is a leading cause of mortality and disability worldwide, it ranks second among global mortality contributors and third among causes of combined disability and death, highlighting its status as a critical public health priority.^[1]^ Beyond its high mortality rate, the disabling consequences of stroke are significant; nearly half of survivors experience permanent functional impairments, including hemiplegia, swallowing difficulties, and speech disorders.^[2]^ The global incidence of stroke-related disability has risen by 32%, resulting in prolonged physical and mental suffering for patients and imposing a considerable strain on family caregivers and social healthcare resources.^[3,4]^ Globally, the stroke burden is anticipated to rise by 81% by 2050. Without timely and effective interventions, stroke-related deaths may rise by 50%, and the cumulative economic loss could reach $2.3 trillion.^[5]^ In this context, primary prevention has been identified as a crucial strategy for reducing stroke incidence, emphasizing the effective management of underlying diseases and the adoption of healthy lifestyles.^[6,7]^ Among the various modifiable lifestyle factors, dietary patterns have garnered significant attention in recent years. Research has confirmed that diet not only directly affects stroke risk^[8]^ but also indirectly influences it by regulating major stroke risk factors, such as blood pressure, blood sugar, and blood lipids.^[9]^ Numerous cross-sectional and cohort studies have validated the association between healthy dietary habits and reduced stroke risk.^[10]^ Consequently, exploring the mechanisms linking diet and stroke in depth can provide an evidence-based foundation for stroke prevention and is of great significance in mitigating the global burden of stroke.

Recent epidemiological studies have consistently demonstrated a significant correlation between dietary habits and the risk of stroke. A systematic review and meta-analysis of 15 prospective cohort studies revealed that the consumption of total, red, and processed meat is significantly associated with an elevated risk of stroke, whereas the intake of white meat appears to have a protective effect.^[11]^ However, this analysis did not establish a significant link between meat consumption and stroke mortality, and it lacked sufficient examination of the specific impacts of various stroke subtypes. A European cohort study indicated that replacing red and processed meat with plant protein may decrease stroke risk, and that dietary patterns exhibit subtype-specific associations; the consumption of dairy products, fruits and vegetables, and dietary fiber is linked to a reduced risk of ischemic stroke, whereas egg consumption is associated with an increased risk of hemorrhagic stroke.^[12]^ Additionally, a prospective study from the UK Biobank suggested that vegetable intake may mitigate the stroke mortality risk associated with environmental pollutants. Notably, raw vegetable consumption is linked to a reduced risk of cardiovascular disease, including stroke, whereas cooked vegetables do not demonstrate similar effects, and residual confounding factors may significantly influence the reliability of these associations.^[13]^

Despite these research advancements, significant limitations persist in the current literature. Primarily, the investigation of stroke subtypes remains predominantly confined to the broad categories of ischemic and hemorrhagic strokes. There is a notable paucity of comprehensive studies focusing on subdivisions such as lacunar strokes and small vessel ischemic strokes, and the distinct effects of various dietary factors on these subtypes remain inadequately understood. Furthermore, most recent studies employed observational designs, rendering them vulnerable to confounding due to collinearity between dietary habits and lifestyle factors, as well as reverse causation, thereby complicating the establishment of causal relationships. Although randomized controlled trials represent the optimal approach to overcoming the limitations inherent to observational studies, ethical considerations often render such trials impractical. Consequently, there is an urgent need for research methodologies capable of effectively controlling confounding variables and inferring causal associations to address these gaps. Mendelian randomization (MR) is an analytical method rooted in statistical genetics, which uses genetic variants as instrumental variables (IVs) to verify the causal relationship between genetically predicted exposures and clinical endpoints of interest. Single nucleotide polymorphisms (SNPs), the core genetic markers used in this method, are typically obtained from large, publicly available genome-wide association studies (GWAS). Based on the natural randomization of genetic variants at the time of conception, this approach can significantly reduce the impact of confounding factors and effectively avoid the reverse causation bias that is common in traditional observational research. Previous MR analyses in this domain have concentrated on mediating factors, have not encompassed all stroke subtypes, and have not conducted reverse MR analysis to exclude potential reverse causation.^[14]^

This study utilized a two-sample MR analysis to systematically assess the causal relationship between genetically predicted dietary factors and stroke, including its subtypes. This approach aims to provide more reliable evidence-based support for dietary prevention strategies targeting stroke.

## 2. Methods

### 2.1. Study design

**Figure 1.**
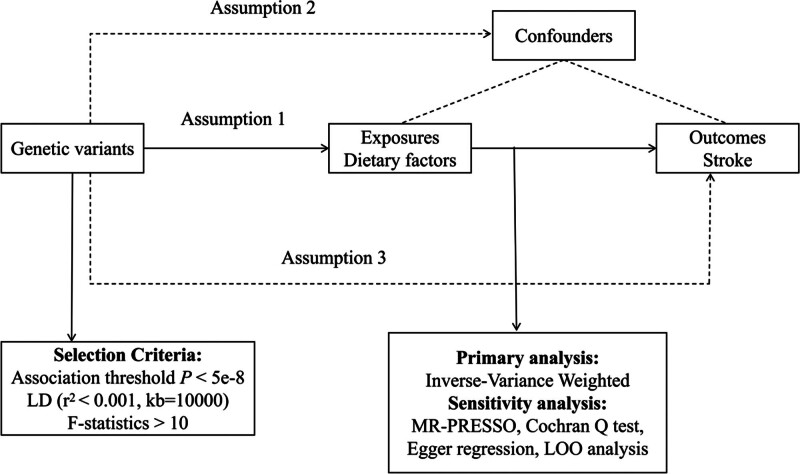
Overview of the MR study design and methods. LD = linkage disequilibrium; LOO = leave-one-out; MR = Mendelian randomization; MR-PRESSO = Mendelian Randomization Pleiotropy RESidual Sum and Outlier.

To assess the potential causal relationship between genetically predicted dietary factors and stroke risk, we first employed a univariable two-sample MR design (Fig. 1). The validity of MR analysis relies on 3 key assumptions: relevance: the selected genetic IVs are strongly associated with the target dietary factors; independence: these IVs are not related to any confounding variables that could influence the exposure-outcome relationship; exclusion restriction: the IVs affect stroke risk only through their association with dietary factors. To rule out the possibility of reverse causality, we conducted a reverse MR analysis in which stroke was treated as the exposure and dietary factors as the outcome.

### 2.2. Data sources

All SNPs required for this study were obtained from different sample sets and studies of the same ethnic population. We extracted genetic instruments corresponding to each variable from the IEU OpenGWAS (https://gwas.mrcieu.ac.uk/) and the GWAS catalog (https://www.ebi.ac.uk/gwas/). Specifically, summary data on SNPs related to dietary intake were sourced from the MRC Integrative Epidemiology Unit Consortium based on the UK Biobank. The specific types of dietary intake are listed in Table S1, Supplemental Digital Content, https://links.lww.com/MD/R696, including dried fruit, oily fish, processed meat, salad/raw vegetable, nonoily fish, fresh fruit, beef, pork, cooked vegetable, poultry, lamb/mutton, and coffee intakes, for a total of 12 categories. The outcome indicators included stroke and its subtypes (including ischemic stroke, ischemic stroke [small vessel], lacunar stroke, intracerebral hemorrhage [ICH], and subarachnoid hemorrhage) (Table S2, Supplemental Digital Content, https://links.lww.com/MD/R696).

All GWAS involved in this study were conducted exclusively on individuals of European ancestry, and the exposure data from the UK Biobank were independent of the outcome data from FinnGen and the GWAS catalog. Every original GWAS included in our analysis had secured ethical committee approval prior to its conduct, with informed consent duly collected from all study participants. As such, our research, which relies on the secondary analysis of de-identified public GWAS summary statistics, does not require any additional ethical approval.

### 2.3. Genetic instrument selection

To meet the requirements of univariate two-sample MR analysis, this study adopted a standardized procedure to screen genetic IVs for the “dietary factors (exposure)–stroke (outcome)” relationship. SNPs associated with dietary factors were extracted as initial candidate IVs from relevant GWAS datasets, using a genome-wide significance of *P* < 5 × 10^−8^ as the initial candidate IVs. Linkage disequilibrium pruning was performed using a 10,000 kb window and an *r*^2^ < 0.001 threshold to remove redundant SNPs and ensure independence (Fig. 1). The LDtrait tool was used to exclude confounding SNPs associated with stroke, and palindromic SNPs were removed to avoid bias. The remaining SNPs were extracted from the stroke GWAS dataset, and proxy SNPs with *r*^2^ > 0.001 were used for alignment if the original SNPs were unavailable. Finally, the *F* statistics were calculated, and weak instruments with *F* < 10 were excluded.

### 2.4. Statistical analysis

The inverse-variance weighted (IVW) method was used as the primary model for the MR analysis. This method calculates the Wald ratio for each genetic instrumental variable SNP and integrates the effect estimates of each SNP based on the principles of a meta-analysis. It offers high statistical power and is the most widely used core method for MR analysis. The causal effect was expressed as an odds ratio (*OR*) and 95% confidence interval (95% *CI*), which quantifies the change in disease risk for each 1 standard deviation (SD) increase in dietary factors.

To verify the robustness of the causal inference results, this study conducted multiple sensitivity analyses: Cochran *Q* test: used to assess the heterogeneity of effect sizes among SNPs. If the test result *P* < .05, it indicates significant heterogeneity; MR-Egger regression detects potential horizontal pleiotropy by examining the intercept of the regression. If the intercept significantly deviated from 0 (*P* < .05), it suggested the presence of pleiotropic bias; Mendelian Randomization Pleiotropy RESidual Sum and Outlier (MR-PRESSO) method: used to identify and correct the influence of outlier SNPs on the overall effect estimate, thereby reducing the bias caused by outliers. If *P* < .05, it indicates that there are significant outliers or horizontal pleiotropy in the current MR analysis; leave-one-out analysis: sequentially excludes each individual SNP and recalculates the MR analysis and effect estimate to evaluate whether any single SNP plays a dominant role in the overall causal estimate. Funnel, forest, and scatter plots were used for the visual analysis. All statistical analyses were performed using R software (version 4.4.3; R Foundation for Statistical Computing, Vienna, Austria), with core MR analyses mainly performed using the “TwoSampleMR” package.

## 3. Results

### 3.1. MR analysis

**Figure 2.**
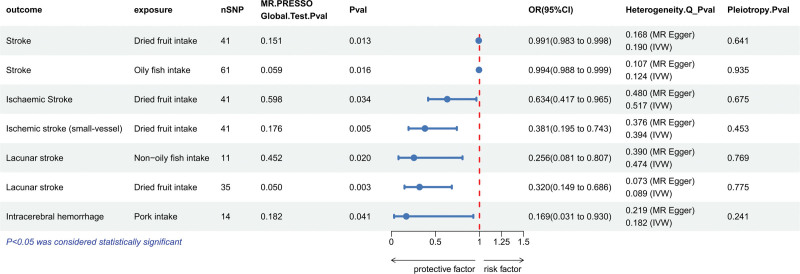
MR analysis of the associations between dietary exposure and disease. This figure only visually summarizes the statistically significant associations identified in the Mendelian randomization analysis. The complete set of results, including nonsignificant associations, are detailed in Table S4 of the supplementary data. MR = Mendelian randomization.

A total of 12 exposure factors were enrolled in the present Mendelian randomization analysis. After rigorous sequential quality control screening, the number of SNPs finally included for each exposure variable varied between 7 and 61, with full details presented in Table S3, Supplemental Digital Content, https://links.lww.com/MD/R696. The instrumental strength of all included genetic IVs was considered fully sufficient, given that the *F*-statistics of each IV in our study were all above the critical threshold of 10. Figure 2 and Table S4, Supplemental Digital Content, https://links.lww.com/MD/R696 present the results of the two-sample MR analysis of dietary factors and stroke risk. The results showed that dried fruit intake significantly reduced the risk of stroke (*OR* = 0.991, 95% *CI*: 0.983–0.998, *P* = .0127) and its subtypes: ischemic stroke (*OR* = 0.634, 95% *CI*: 0.417–0.965, *P* = .0337), ischemic stroke (small-vessel) (*OR* = 0.381, 95% *CI*: 0.195–0.743, *P* = .0046), and lacunar stroke (*OR* = 0.320, 95% *CI*: 0.149–0.686, *P* = .0034). Oily fish intake also appeared to be a protective factor against stroke (*OR* = 0.994, 95% *CI*: 0.988–0.999; *P* = .0162). Non-oily fish intake significantly reduced the risk of lacunar stroke (*OR* = 0.256, 95% *CI*: 0.081–0.807, *P* = .0200). Pork intake notably reduced the risk of ICH (*OR* = 0.169, 95% *CI*: 0.031–0.930, *P* = .0410). No significant causal associations were detected between processed meat, salad/raw vegetables, fresh fruit, beef, cooked vegetables, poultry, lamb/mutton, and coffee intakes and stroke or its subtypes in the IVW and other supplementary MR methods (all *P*-value for all methods >.05).

### 3.2. Sensitivity analysis

We conducted sensitivity analyses to verify the robustness of MR results. Figure 2 shows that the *P*-value for MR-PRESSO, Cochran *Q* test, Egger regression, and other testing methods for variables with causal associations in the MR results were all ≥0.05. The scatter plot (Fig. S1, Supplemental Digital Content, https://links.lww.com/MD/R695) demonstrates a significant negative causal relationship between dietary factors with causal associations and disease. The MR estimates are presented in the forest plot (Fig. S2, Supplemental Digital Content, https://links.lww.com/MD/R695), where the IVW regression coefficients for all statistically significant protective associations were negative. According to the results of the leave-one-out analysis, the overall estimates remained consistent after each exclusion (Fig. S3, Supplemental Digital Content, https://links.lww.com/MD/R695). Similarly, our funnel plot-based estimates lacked evidence of horizontal pleiotropy (Fig. S4, Supplemental Digital Content, https://links.lww.com/MD/R695).

### 3.3. Reverse MR analysis

We used the same method as in the forward analysis to perform a reverse MR analysis of the association between stroke and the dietary variables. Overall, no widespread and robust reverse causal associations were detected. Only 2 nominally significant associations were observed under the primary IVW method. First, genetic predisposition to stroke was associated with decreased oily fish intake (IVW: *OR* = 0.366, 95% *CI :* 0.159–0.839, *P* = .016), while this association was not significant in MR Egger, weighted median, simple mode, or weighted mode analyses (all *P* > .05), indicating poor robustness of this signal. Second, genetic predisposition to lacunar stroke was associated with decreased dried fruit intake (IVW: *OR* = 0.993, 95% *CI :* 0.988–0.999, *P* = .016). Only the weighted median method showed a consistent nominally significant result (*P* = .026), with no significance in the other 3 supplementary methods (all *P* > .05), and the effect size was extremely small, with no meaningful clinical relevance. (Table S5, Supplemental Digital Content, https://links.lww.com/MD/R696).

## 4. Discussion

In this study, we performed a two-sample MR analysis to investigate the causal associations between 12 distinct dietary exposures and the risk of stroke. These findings suggest that dried fruit intake conferred a significant protective effect against stroke, ischemic stroke, ischemic stroke (small-vessel), and lacunar stroke. Additionally, nonoily fish intake demonstrated a protective effect against lacunar stroke, whereas oily fish consumption was associated with a reduced risk of stroke. Furthermore, higher pork intake was linked to a reduced risk of ICH. No significant causal associations were detected between the remaining included dietary factors and stroke risk.

Dried fruits are concentrated food products derived from fresh fruits through natural air drying or artificial dehydration, which typically reduces the moisture content to below 15%. While the drying process may lead to the loss of certain water-soluble vitamins, such as vitamin C, it significantly concentrates numerous essential nutrients, including minerals, dietary fiber, and phenolic antioxidants, such as flavonoids and anthocyanins, per unit weight. This process enhances the nutritional density and functional properties of dried fruits.^[15]^ The concentration effect enhances the health benefits of dried fruits. For instance, dried jujubes are abundant in potassium and magnesium, and the nutritional and functional attributes of seedless raisins are effectively preserved post-drying.^[16,17]^ Furthermore, dried fruits offer practical advantages, such as low moisture content, extended shelf life, and ease of transportation and storage, rendering them convenient nutritional supplements in daily diets. Emerging drying technologies, such as radio frequency vacuum drying, further optimize nutrient and flavor retention.^[18]^

In the MR analysis conducted in this study, the IVW method was employed to examine data on dietary exposure levels predicted by genetic IVs. The results indicated a significant negative correlation between dried fruit consumption and the overall risk of stroke, ischemic stroke, small-vessel ischemic stroke, and lacunar stroke. This suggests that dried fruit intake may be a protective factor against cerebrovascular diseases. Conversely, this study did not identify any statistically significant association between the consumption of fresh fruits or raw/cooked vegetables and the risk of stroke.

Epidemiological modeling studies have demonstrated that substituting unhealthy snacks with dried fruit can substantially decrease stroke mortality by reducing salt and saturated fat intake, potentially lowering approximately 425 cases annually.^[19]^ Experimental research has shown that bioactive components in dried fruit, such as osthol, can enhance lipid metabolism and mitigate the risk of atherosclerosis by inhibiting blood pressure elevation, regulating 3-hydroxy-3-methylglutaryl coenzyme A reductase activity, and promoting fatty acid oxidation.^[20]^ Furthermore, traditional nut-based medicines have exhibited neuroprotective potential in ischemic stroke models through multi-target mechanisms, including the inhibition of oxidative stress and inflammatory responses and regulation of neurovascular unit function.^[21]^ These findings align with the genetic evidence from this study and collectively suggest that dried fruit may reduce the risk of stroke by improving cardiovascular and metabolic indicators and providing direct protection to the brain. Notably, the lack of a protective effect of fresh fruits and vegetables against stroke warrants further investigation in future studies. Considering the nutritional properties of fresh fruits and vegetables, a potential explanatory factor is that their elevated water content results in a substantially lower concentration of bioactive components associated with stroke prevention per unit weight compared to dried fruits. This reduced nutrient density may present a challenge in reaching the effective intake threshold of these bioactive substances through daily consumption. Nonetheless, direct evidence supporting this dose–response relationship in the context of stroke prevention remains limited and necessitates further investigation and validation. Additionally, the significant heterogeneity among fruit and vegetable types (e.g., substantial compositional differences between leafy greens and melons) may obscure the association signals of specific subtypes, and recall bias in dietary assessment may also affect the accuracy of our results. This suggests that future research should refine fruit and vegetable classifications, optimize intake assessment methods, and validate associations using biomarkers.

Oily fish are characterized by a high fat content, typically exceeding 5%, and are rich in long-chain omega-3 polyunsaturated fatty acids, primarily eicosapentaenoic acid and docosahexaenoic acid. Common examples include salmon, mackerel, sardines, herring, and trout.^[22]^ In contrast, nonoily fish have a lower fat content, generally <5%, and are high in protein, easily digestible, and provide omega-3 polyunsaturated fatty acids and vitamins B12 and B6. Typical nonoily fish include cod, pollock, halibut, tilapia, and water-packed tuna.^[23]^ The cardiovascular protective effects of oily fish have been extensively investigated, primarily attributed to their high concentrations of long-chain omega-3 polyunsaturated fatty acids.^[22]^ These omega-3 fatty acids are metabolized into compounds such as resolvins and protectins, which exhibit potent anti-inflammatory properties, effectively stabilize arterial plaques, prevent plaque rupture that could lead to thrombosis, and ultimately reduce the risk of ischemic stroke. Additionally,omega-3 fatty acids are often considered to have potential antithrombotic effects, as evidenced by the extended bleeding times observed at very high dosages (e.g., 15 g/day).^[24]^ Furthermore, these components support the health of vascular endothelial cells and promote vasodilation, ensuring unobstructed blood flow. A prospective cohort study demonstrated that, versus a weekly fish consumption of <300 g, an intake of 300 to 450 g per week was associated with a significantly reduced risk of both total stroke (HR: 0.78, 95% CI: 0.64–0.94) and ischemic stroke (HR: 0.70 [0.57–0.88]).^[25]^ Another meta-analysis has identified a linear dose–response association of fish consumption with stroke risk, with a 2% to 12% reduction in stroke incidence as weekly fish intake increased from 100 to 700 g.^[26]^ A large prospective study also indicated that higher levels of omega-3 polyunsaturated fatty acids were associated with a lower risk of total and ischemic stroke but not hemorrhagic stroke, which is consistent with the findings of the current study.

Recent studies have suggested that a high intake of red and processed meat is associated with an elevated risk of cardiovascular disease.^[27]^ The proposed biological pathways for this observed correlation mainly involve the adverse effects of red meat intake on traditional cardiovascular risk factors: for example, a randomized controlled trial in healthy populations found that long-term red meat intake could significantly elevate serum low-density lipoprotein cholesterol levels compared with a vegetarian diet.^[28]^ Meanwhile, red meat consumption is also closely linked to the onset and progression of obesity,^[29]^ a well-established independent risk factor for both ischemic and hemorrhagic stroke. Another plausible explanation for the positive correlation between red meat intake and cardiovascular disease is the presence of certain fatty acids, such as arachidonic acid, which have been shown to increase the risk of cardiovascular disease.^[30]^ However, it should be emphasized that the above findings are all derived from observational study designs, which can only reveal correlation rather than definitive causal relationship, and the causal inference is inevitably limited by the inherent defects of such study designs. Most previous studies adopted an observational design, which is difficult to completely eliminate the interference of confounding factors, even after multivariate adjustment. These potential confounders include unhealthy lifestyle habits, overall dietary patterns, socioeconomic status, and other concurrent health-related behaviors, which may lead to residual confounding and ultimately biased or unstable results. In contrast, the MR design used in this study uses genetic variants as instrumental variables for dietary exposure, which can minimize the interference of confounding factors and reverse causality at the genetic level, thus providing more robust evidence for causal inference. A large cohort study indicated that animal product intake had a protective effect against ICH but was not related to mortality from cerebral infarction.^[31]^ Notably, prior Mendelian randomization studies exploring the association between consumption of processed and red meat and cardiovascular events^[32,33]^ failed to identify a significant causal relationship between these factors. These findings are highly consistent with those of our present study, which further confirms that processed and red meat intake does not exert a significant pro-risk effect on overall stroke incidence. This consistent finding further supports the reliability of our study’s conclusions. However, another cohort study^[34]^ found that red meat intake was associated with a reduced risk of death due to hemorrhagic stroke. This aligns with our findings, as we observed that pork (a primary type of red meat) was associated with a reduced risk of ICH, a relationship that has not been previously reported in MR studies. Additionally, a basic research study demonstrated that certain bioactive peptides extracted from pork (pork peptides) exhibit antithrombotic activity, possibly by inhibiting platelet aggregation and reducing thrombosis.^[35]^ This finding may further elucidate our observed results through the “prevention of atherosclerotic thrombosis” pathway.

Several limitations of this study should be acknowledged. This study only included 12 common dietary factors, and did not cover other dietary components or different food processing and cooking methods (such as frying, roasting, and marinating), which may lead to the failure to distinguish the heterogeneous effects of different dietary treatment modalities on stroke subtypes. Additionally, the absence of GWAS data on food preparation methods, such as frying or roasting, may have impeded our ability to differentiate the effects of various cooking methods on stroke subtypes. All genome-wide association study summary data included in this work were sourced exclusively from European ancestry populations, which may limit the generalizability of the findings to other ethnic groups, such as East Asians or individuals of African descent. Furthermore, the study did not account for potential confounding factors, including socioeconomic status and cultural dietary habits, which further restricts the extrapolation of the conclusions to other populations. Although this study employed methods such as MR-PRESSO to detect horizontal pleiotropy, unmeasured pleiotropy may persist. Some genetic variants may concurrently influence dietary preferences and stroke risk, and current methodologies cannot eliminate such confounding factors. Moreover, the core assumptions underlying MR analysis cannot be fully verified, potentially affecting the robustness of causal inference. Meanwhile, the relatively small sample size of some rare stroke subtypes (such as subarachnoid hemorrhage) may lead to insufficient statistical power to detect weak causal effects. Nevertheless, we have adopted strict genetic instrument screening criteria and multidimensional sensitivity analyses to minimize the above biases, and the results of this study still provide more reliable genetic evidence for the causal association between dietary factors and stroke compared with previous observational studies.

Reverse MR analyses were conducted to mitigate concerns of reverse causation bias, a notable study strength. While no pervasive reverse causal associations emerged between genetic stroke predisposition and dietary intake, 2 nominally significant reverse signals were observed: genetic risk for stroke was inversely associated with oily fish intake (IVW: *P* = .016), yet this finding lacked replication in supplementary MR methods, suggesting a statistical artifact rather than true bidirectionality. Similarly, genetic predisposition to lacunar stroke was linked to reduced dried fruit intake (IVW: *P* = .016); however, this association had a negligible effect size (*OR* = 0.993) and likely reflected a false positive due to large sample size, contrasting with the robust protective effect of dried fruit intake on lacunar stroke in forward MR (*OR* = 0.320, *P* = .003). Collectively, these findings support the validity of forward causal inferences, indicating that dietary effects on stroke are unlikely to be confounded by reverse causality, despite these limited nominal reverse associations.

## 5. Conclusions

In this work, we employed a two-sample MR design to systematically evaluate the potential causal associations between 12 dietary factors and stroke subtypes (ischemic, lacunar, and ICH). We identified several robust protective associations from our analyses: higher dried fruit intake was associated with a reduced risk of both total stroke and ischemic stroke, oily fish intake conferred a protective effect against total stroke, nonoily fish intake was found to decrease the risk of lacunar stroke, and increased pork intake corresponded to a lower risk of ICH. The “potential cumulative effects” of processed and non-pork red meat remain uncertain, which may affect the generalizability of the conclusions. Future research should quantify meat consumption characteristics, including weekly frequency, per-meal intake, and dietary patterns, to better assess their impact on stroke risk. Reverse MR analyses revealed no widespread reverse causal bias for most dietary–stroke associations, supporting the overall robustness of our causal inferences. However, a potential bidirectional association was observed between oily fish intake and stroke risk, and a statistically significant but biologically negligible reverse signal was detected between lacunar stroke and dried fruit intake. These findings suggest that the causal relationships should be interpreted with caution. Collectively, our two-sample MR analysis provides genetic-level causal evidence for the associations between diverse dietary factors and the risk of stroke and its clinical subtypes. Future studies should examine the mechanisms of “protective diets (such as dried fruit, fish, and pork),” to provide scientific support for dietary intervention strategies in primary stroke prevention and help formulate targeted dietary guidelines for stroke prevention.

## Acknowledgments

All authors express their gratitude to the investigators and participants of the original genome-wide association studies, as well as the developers of the R packages utilized in this work. We also sincerely thank the reviewers and editors for their constructive feedback and insightful comments on this manuscript.

## Author contributions

**Conceptualization:** Zhibo Xuan, Huasen Yang, Lining Duan.

**Data curation:** Zhibo Xuan, Huasen Yang, Lining Duan.

**Formal analysis:** Zhibo Xuan, Huasen Yang, Lining Duan.

**Funding acquisition:** Weiwei Shan.

**Investigation:** Mengwan Hu, Weiwei Shan.

**Methodology:** Zhibo Xuan, Huasen Yang, Mengwan Hu.

**Project administration:** Zhibo Xuan, Mengwan Hu, Weiwei Shan.

**Resources:** Zhibo Xuan, Xian Wu.

**Supervision:** Mengwan Hu, Xian Wu, Weiwei Shan.

**Writing – original draft:** Zhibo Xuan, Huasen Yang.

**Writing – review & editing:** Lining Duan, Mengwan Hu, Xian Wu, Weiwei Shan.

## Supplementary Material




