# Supplementary material for: Exploring causal links between multifaceted dietary exposures and stroke subtypes: Results from a two-sample Mendelian randomization analysis
Source: Medicine (Baltimore). 2026 Apr 17;105(16):e48375. doi: 10.1097/MD.0000000000048375 (PMC13095255; doi:10.1097/MD.0000000000048375)
Supplement: Supplementary file 1 [file medi-105-e48375-s001.pdf]

Table.S1 Summary information of dietary intake exposures

Trait	GWAS data	Sample size
Dried fruit intake	ukb-b-16576	421764
Oily fish intake	ukb-b-2209	460443
Processed meat intake	ukb-b-6324	461981
Salad/raw vegetable intake	ukb-b-1996	435435
Non-oily fish intake	ukb-b-17627	460880
Fresh fruit intake	ukb-b-3881	446462
Beef intake	ukb-b-2862	461053
Pork intake	ukb-b-5640	460162
Cooked vegetable intake	ukb-b-8089	448651
Poultry intake	ukb-b-8006	461900
Lamb/mutton intake	ukb-b-14179	460006
Coffee intake	ukb-b-5237	428860

Table.S2 GWAS data for stroke and subtypes

Trait	GWAS data	Cases	Controls
Stroke	ebi-a-GCST90038613	6925	477,673
Ischaemic Stroke, excluding all haemorrhages	finn-b-I9_STR_EXH	10551	202223
Ischemic stroke (small-vessel)	ebi-a-GCST006909	5386	192662
Lacunar stroke	ebi-a-GCST90014122	6030	248929
Intracerebral hemorrhage	ebi-a-GCST90018870	1935	471578
Subarachnoid hemorrhage	ebi-a-GCST90018923	1693	471562

Table.S3 Information of genetic instrumental variants for dietary intake

Trait	SNP	chr	pos	EA	OA	eaf	beta	se	pval	R2	F	samplesize
Coffee intake	rs2472297	15	75,027,880	T	C	0.262883	0	0.00182733	1.099E-142	0.00083693	359	428860
Coffee intake	rs4410790	7	17284577	C	T	0.632141	0.039072	0.00167288	1.1995E-120	0.000709997	304.7043108	428860
Coffee intake	rs1421085	16	53800954	C	T	0.40357	0.0185426	0.00164436	1.69981E-29	0.00016552	70.99618564	428860
Coffee intake	rs1057868	7	75615006	T	C	0.284986	0.0199509	0.00178517	5.40008E-29	0.000162216	69.57881395	428860
Coffee intake	rs476828	18	57852587	C	T	0.237409	0.0173461	0.00189543	5.60015E-20	0.000108949	46.72866521	428860
Coffee intake	rs780093	2	27742603	C	T	0.616	0.013	0.002	1.000E-15	0.00008	35.862	428860
Coffee intake	rs34060476	7	73037956	G	A	0.134	0.018	0.002	7.501E-15	0.00008	33.777	428860
Coffee intake	rs56113850	19	41353107	C	T	0.578	0.013	0.002	8.900E-15	0.00008	33.567	428860
Coffee intake	rs13387939	2	637498	A	C	0.828	0.017	0.002	9.799E-15	0.00008	33.428	428860
Coffee intake	rs13163336	5	87943710	A	C	0.158	0.015	0.002	1.300E-11	0.00006	25.444	428860
Coffee intake	rs61928609	12	11316437	C	A	0.835	-0.015	0.002	1.300E-11	0.00006	25.602	428860
Coffee intake	rs630194	18	40950954	C	T	0.343	-0.011	0.002	2.300E-11	0.00006	24.929	428860
Coffee intake	rs12514566	5	7391462	A	G	0.337	-0.011	0.002	2.400E-11	0.00006	24.899	428860
Coffee intake	rs17842490	22	24870527	G	A	0.014	-0.045	0.007	3.300E-11	0.00006	24.579	428860
Coffee intake	rs7224815	17	17845800	T	A	0.408	-0.011	0.002	3.700E-11	0.00006	24.433	428860
Coffee intake	rs4615895	1	96274668	A	G	0.741	0.012	0.002	4.200E-11	0.00006	24.517	428860
Coffee intake	rs73075167	7	17570479	T	A	0.129	-0.016	0.002	5.000E-11	0.00006	24.900	428860
Coffee intake	rs6062682	20	62891820	T	C	0.465	0.010	0.002	2.500E-10	0.00005	22.946	428860
Coffee intake	rs6063085	20	45840459	C	A	0.373	0.010	0.002	4.500E-10	0.00005	21.753	428860
Coffee intake	rs2465037	6	51179260	A	C	0.343	-0.011	0.002	4.800E-10	0.00005	21.849	428860

Trait	SNP	chr	pos	EA	OA	eaf	beta	se	pval	R2	F	samplesize
Coffee intake	rs9398171	6	108983527	T	C	0.711	0.011	0.002	1.100E-09	0.00005	20.794	428860
Coffee intake	rs8056750	16	70927078	T	C	0.359	0.011	0.002	1.300E-09	0.00005	21.904	428860
Coffee intake	rs2189234	4	106075498	G	T	0.618	0.010	0.002	1.800E-09	0.00005	20.201	428860
Coffee intake	rs442355	8	109128653	C	G	0.254	-0.011	0.002	1.900E-09	0.00005	20.183	428860
Coffee intake	rs78267637	8	33790200	G	C	0.038	-0.025	0.004	3.900E-09	0.00005	20.330	428860
Coffee intake	rs516636	1	177855517	A	C	0.209	0.012	0.002	4.000E-09	0.00005	19.328	428860
Coffee intake	rs13054099	22	41215672	C	T	0.261	-0.011	0.002	4.300E-09	0.00004	19.218	428860
Coffee intake	rs1338549	6	98312143	G	T	0.534	-0.009	0.002	5.600E-09	0.00004	19.067	428860
Coffee intake	rs117810762	10	135315795	A	G	0.018	0.036	0.006	6.200E-09	0.00005	19.423	428860
Coffee intake	rs57918684	17	60150383	A	G	0.155	0.013	0.002	8.600E-09	0.00004	18.631	428860
Coffee intake	rs10119174	9	27953724	C	G	0.571	-0.009	0.002	1.000E-08	0.00004	18.557	428860
Coffee intake	rs1527961	2	62780440	C	T	0.135	-0.013	0.002	1.700E-08	0.00004	17.822	428860
Coffee intake	rs6469262	8	110443480	C	T	0.565	-0.009	0.002	1.900E-08	0.00004	17.664	428860
Coffee intake	rs117968677	15	75174251	A	G	0.024	-0.031	0.006	1.900E-08	0.00005	19.508	428860
Coffee intake	rs2597805	4	17424930	T	C	0.682	0.010	0.002	2.000E-08	0.00004	18.053	428860
Coffee intake	rs75347775	19	18495908	A	G	0.245	0.010	0.002	2.700E-08	0.00004	17.305	428860
Coffee intake	rs12989746	2	49368391	T	G	0.250	0.010	0.002	2.800E-08	0.00004	17.225	428860
Coffee intake	rs1942965	18	55032486	C	T	0.505	-0.009	0.002	3.800E-08	0.00004	16.997	428860
Coffee intake	rs7811609	7	32930597	T	C	0.375	0.009	0.002	4.000E-08	0.00004	16.785	428860
Coffee intake	rs2107308	17	46164947	T	A	0.245	-0.010	0.002	4.100E-08	0.00004	16.834	428860

Trait	SNP	chr	pos	EA	OA	eaf	beta	se	pval	R2	F	samplesize
Salad / raw vegetable intake	rs7619139	3	25110415	A	T	0.589	0.012	0.001	8.000E-18	0.00008	32.814	435435
Salad / raw vegetable intake	rs790561	8	64618026	G	A	0.704	0.012	0.002	1.400E-15	0.00006	28.232	435435
Salad / raw vegetable intake	rs3095337	6	30737591	C	G	0.204	-0.013	0.002	8.999E-13	0.00005	22.522	435435
Salad / raw vegetable intake	rs6482190	10	22037809	G	A	0.719	0.011	0.002	1.400E-12	0.00005	22.337	435435
Salad / raw vegetable intake	rs17460017	5	137717681	T	A	0.190	0.011	0.002	7.200E-10	0.00004	16.744	435435
Salad / raw vegetable intake	rs62461186	7	77730153	C	A	0.180	-0.011	0.002	1.000E-09	0.00004	16.529	435435
Salad / raw vegetable intake	rs12203592	6	396321	T	C	0.219	-0.010	0.002	1.300E-09	0.00004	15.778	435435
Salad / raw vegetable intake	rs10819082	9	128645617	A	G	0.667	-0.009	0.002	1.400E-09	0.00004	16.239	435435
Salad / raw vegetable intake	rs2194027	5	87822672	A	T	0.485	-0.009	0.001	2.000E-09	0.00004	16.118	435435
Salad / raw vegetable intake	rs7227822	18	38107825	G	C	0.518	-0.008	0.001	3.700E-09	0.00004	15.346	435435
Salad / raw vegetable intake	rs57221424	7	35215670	G	C	0.322	0.009	0.002	5.500E-09	0.00003	15.193	435435
Salad / raw vegetable intake	rs1890012	13	54933682	G	T	0.195	-0.010	0.002	8.100E-09	0.00003	14.844	435435
Salad / raw vegetable intake	rs1052352	16	31195279	T	C	0.524	0.008	0.001	1.000E-08	0.00003	14.481	435435
Salad / raw vegetable intake	rs12908495	15	97011280	A	C	0.243	-0.009	0.002	2.000E-08	0.00003	13.987	435435
Salad / raw vegetable intake	rs75248709	6	92348945	T	C	0.046	-0.020	0.004	2.200E-08	0.00003	14.867	435435
Salad / raw vegetable	rs1828021	4	63740518	G	A	0.499	0.008	0.001	2.400E-08	0.00003	13.880	435435

Trait	SNP	chr	pos	EA	OA	eaf	beta	se	pval	R2	F	samplesize
intake												
Salad / raw vegetable intake	rs9427220	1	15376175 0	T	A	0.555	-0.008	0.001	2.800 E-08	0.00003	13.795	435435
Salad / raw vegetable intake	rs8130508	21	19049865	A	G	0.290	0.009	0.002	3.000 E-08	0.00003	13.690	435435
Salad / raw vegetable intake	rs4083969	1	23186648 0	G	C	0.057	0.017	0.003	3.800 E-08	0.00003	13.790	435435
Salad / raw vegetable intake	rs7821179	8	83061099	C	G	0.847	-0.011	0.002	4.400 E-08	0.00003	13.239	435435
Salad / raw vegetable intake	rs34186148	17	43854655	C	G	0.370	-0.008	0.001	4.800 E-08	0.00003	13.161	435435
Oily fish intake	rs11859365	16	83683945	C	A	0.254	0.023	0.002	9.399 E-25	0.00019	88.826	460443
Oily fish intake	rs3124402	13	55975115	G	A	0.733	-0.022	0.002	1.900 E-24	0.00019	87.203	460443
Oily fish intake	rs4002471	19	49215095	T	C	0.547	-0.019	0.002	1.500 E-23	0.00018	84.508	460443
Oily fish intake	rs10828250	10	21847178	G	C	0.309	-0.020	0.002	2.600 E-22	0.00017	79.866	460443
Oily fish intake	rs1421085	16	53800954	C	T	0.403	0.018	0.002	2.500 E-21	0.00016	75.713	460443
Oily fish intake	rs35287743	12	11005725 0	T	G	0.116	-0.028	0.003	7.000 E-21	0.00016	75.141	460443
Oily fish intake	rs16891727	6	26488860	A	C	0.130	-0.024	0.003	6.800 E-17	0.00013	58.523	460443
Oily fish intake	rs1876245	3	71534763	C	T	0.431	0.015	0.002	5.000 E-15	0.00011	51.623	460443
Oily fish intake	rs11767283	7	12194745 6	G	A	0.222	0.018	0.002	2.500 E-14	0.00011	49.639	460443
Oily fish intake	rs9606833	22	31750013	C	T	0.244	0.017	0.002	2.700 E-14	0.00011	48.959	460443
Oily fish intake	rs28533540	15	47867762	A	G	0.534	0.015	0.002	2.800 E-14	0.00011	49.132	460443
Oily fish intake	rs11986122	8	10009949	G	C	0.423	0.015	0.002	2.900 E-14	0.00011	49.339	460443
Oily fish intake	rs9841174	3	16718487 8	C	T	0.374	0.015	0.002	8.500 E-14	0.00010	47.086	460443
Oily fish	rs3455	6	2609	T	G	0.098	-0.024	0.003	1.500	0.0001	45.914	4604

Trait	SNP	chr	pos	EA	OA	eaf	beta	se	pval	R2	F	samplesize
intake	5420		0270						E-13	0		43
Oily fish intake	rs1951286	14	29766012	G	T	0.645	-0.015	0.002	3.000E-13	0.00010	44.853	460443
Oily fish intake	rs4869859	6	155847549	C	T	0.450	0.014	0.002	3.100E-13	0.00010	44.738	460443
Oily fish intake	rs631490	11	111452040	C	G	0.709	-0.015	0.002	6.001E-13	0.00009	43.544	460443
Oily fish intake	rs45501495	1	204596454	T	C	0.236	0.016	0.002	3.700E-12	0.00009	40.829	460443
Oily fish intake	rs790564	8	64604218	C	A	0.723	0.015	0.002	7.900E-12	0.00009	39.809	460443
Oily fish intake	rs9889161	16	51495068	T	G	0.358	-0.013	0.002	2.800E-11	0.00008	37.552	460443
Oily fish intake	rs4510068	17	44184828	T	G	0.403	-0.013	0.002	4.000E-11	0.00008	37.518	460443
Oily fish intake	rs28623270	17	29389026	T	A	0.149	-0.018	0.003	7.300E-11	0.00008	37.078	460443
Oily fish intake	rs4278546	11	79217391	G	A	0.441	0.013	0.002	9.300E-11	0.00008	35.791	460443
Oily fish intake	rs114497213	3	115214586	T	G	0.055	0.027	0.004	1.100E-10	0.00008	35.617	460443
Oily fish intake	rs12855717	13	101252635	T	C	0.527	-0.012	0.002	2.000E-10	0.00007	34.337	460443
Oily fish intake	rs6465487	7	95760694	G	A	0.400	-0.012	0.002	2.700E-10	0.00007	33.726	460443
Oily fish intake	rs17050031	2	59488019	T	C	0.480	-0.012	0.002	3.500E-10	0.00007	33.374	460443
Oily fish intake	rs13070166	3	85517507	A	T	0.229	0.014	0.002	4.400E-10	0.00007	32.794	460443
Oily fish intake	rs59355765	18	53431951	T	C	0.160	-0.016	0.003	4.700E-10	0.00007	32.706	460443
Oily fish intake	rs303817	12	52176235	G	A	0.751	0.014	0.002	8.000E-10	0.00007	31.747	460443
Oily fish intake	rs10991192	9	107008496	T	C	0.536	-0.012	0.002	1.100E-09	0.00007	31.365	460443
Oily fish intake	rs10513136	3	141107612	A	G	0.065	-0.023	0.004	1.600E-09	0.00007	30.631	460443
Oily fish intake	rs6089753	20	61154107	T	C	0.531	-0.012	0.002	1.800E-09	0.00007	30.554	460443

Trait	SNP	chr	pos	EA	OA	eaf	beta	se	pval	R2	F	samplesize
Oily fish intake	rs12983532	19	18467322	T	C	0.251	-0.013	0.002	2.000E-09	0.00007	31.051	460443
Oily fish intake	rs973526	1	72556393	T	C	0.513	-0.012	0.002	2.500E-09	0.00007	30.464	460443
Oily fish intake	rs2374424	11	83187113	G	A	0.602	-0.011	0.002	4.900E-09	0.00006	28.908	460443
Oily fish intake	rs55985303	2	77224951	A	G	0.241	0.013	0.002	6.600E-09	0.00006	28.361	460443
Oily fish intake	rs275160	2	145967878	C	T	0.701	0.012	0.002	8.000E-09	0.00006	28.371	460443
Oily fish intake	rs61882686	11	46390680	A	C	0.085	0.020	0.003	8.000E-09	0.00006	28.025	460443
Oily fish intake	rs9301837	13	93477312	A	C	0.143	-0.016	0.003	8.100E-09	0.00006	28.016	460443
Oily fish intake	rs6059844	20	33036482	G	A	0.495	0.011	0.002	9.200E-09	0.00006	27.856	460443
Oily fish intake	rs10076975	5	164518993	C	T	0.381	0.011	0.002	1.100E-08	0.00006	27.448	460443
Oily fish intake	rs12663865	6	88103149	A	G	0.758	0.013	0.002	1.100E-08	0.00006	27.543	460443
Oily fish intake	rs9597870	13	59412819	G	T	0.246	-0.013	0.002	1.100E-08	0.00006	27.735	460443
Oily fish intake	rs10510554	3	25099776	C	T	0.569	0.011	0.002	1.200E-08	0.00006	27.546	460443
Oily fish intake	rs9958909	18	1841371	G	T	0.140	0.016	0.003	1.400E-08	0.00006	27.500	460443
Oily fish intake	rs10061973	5	166811498	T	G	0.514	-0.011	0.002	1.500E-08	0.00006	27.093	460443
Oily fish intake	rs7243428	18	35156177	G	A	0.225	-0.013	0.002	1.500E-08	0.00006	27.019	460443
Oily fish intake	rs75887709	19	37438329	G	A	0.136	-0.016	0.003	1.600E-08	0.00006	27.374	460443
Oily fish intake	rs703979	10	80944095	C	T	0.578	0.011	0.002	1.700E-08	0.00006	26.755	460443
Oily fish intake	rs1361016	13	54220229	G	T	0.845	0.015	0.003	1.700E-08	0.00006	27.004	460443
Oily fish intake	rs6033437	20	12495731	A	C	0.257	0.012	0.002	1.700E-08	0.00006	27.361	460443
Oily fish intake	rs7683782	4	14935830	G	C	0.833	0.014	0.003	1.900E-08	0.00006	26.800	460443
Oily fish intake	rs12896749	14	10027949	C	G	0.385	-0.011	0.002	2.500E-08	0.00006	26.206	460443

Trait	SNP	chr	pos	EA	OA	eaf	beta	se	pval	R2	F	samplesize
			2									
Oily fish intake	rs2952140	17	37928059	T	C	0.483	-0.011	0.002	2.500E-08	0.00006	26.216	460443
Oily fish intake	rs9886779	9	15637447	A	T	0.439	-0.011	0.002	2.700E-08	0.00006	26.070	460443
Oily fish intake	rs55930451	2	49039237	T	C	0.108	-0.017	0.003	2.900E-08	0.00006	25.803	460443
Oily fish intake	rs2827161	21	23340050	G	T	0.423	0.011	0.002	3.200E-08	0.00006	25.787	460443
Oily fish intake	rs4982738	14	23650191	A	G	0.583	0.011	0.002	3.500E-08	0.00006	26.411	460443
Oily fish intake	rs905575	3	147076253	G	C	0.824	0.014	0.003	3.600E-08	0.00006	25.743	460443
Oily fish intake	rs7254235	19	22212505	G	A	0.577	-0.011	0.002	4.300E-08	0.00006	25.396	460443
Oily fish intake	rs1201289	3	176822676	G	T	0.395	-0.011	0.002	4.400E-08	0.00005	25.311	460443
Oily fish intake	rs510161	11	28728332	G	C	0.310	-0.011	0.002	4.500E-08	0.00005	25.169	460443
Beef intake	rs9407624	9	15568070	A	T	0.488	-0.014	0.002	1.100E-15	0.00009	43.643	461053
Beef intake	rs10789340	1	72940273	G	A	0.627	-0.014	0.002	6.800E-15	0.00009	40.965	461053
Beef intake	rs4676964	3	71034748	T	C	0.511	0.013	0.002	9.601E-15	0.00009	41.090	461053
Beef intake	rs62396185	6	26180634	C	G	0.260	-0.015	0.002	2.800E-14	0.00008	39.104	461053
Beef intake	rs1421085	16	53800954	C	T	0.403	-0.012	0.002	3.500E-12	0.00007	32.619	461053
Beef intake	rs132901	22	41797547	T	C	0.788	0.014	0.002	2.900E-11	0.00006	29.906	461053
Beef intake	rs429358	19	45411941	C	T	0.154	-0.015	0.002	3.600E-10	0.00006	26.531	461053
Beef intake	rs1105388	1	205308591	T	C	0.300	-0.011	0.002	1.300E-09	0.00005	25.066	461053
Beef intake	rs10959890	9	11526198	C	T	0.212	-0.013	0.002	1.500E-09	0.00005	24.739	461053
Beef intake	rs784251	18	53412903	T	C	0.478	-0.010	0.002	1.700E-09	0.00005	24.593	461053
Beef intake	rs12247907	10	65317045	C	G	0.486	0.010	0.002	8.900E-09	0.00005	22.308	461053
Beef intake	rs1116	1	9774	G	C	0.360	-0.010	0.002	9.800	0.0000	22.104	4610

Trait	SNP	chr	pos	EA	OA	eaf	beta	se	pval	R2	F	samplesize
	5829		3702						E-09	5		53
Beef intake	rs1470610	2	33866464	C	G	0.196	-0.012	0.002	1.500E-08	0.00005	21.701	461053
Beef intake	rs62169335	2	147946069	T	C	0.543	-0.010	0.002	2.400E-08	0.00005	21.468	461053
Beef intake	rs7791463	7	97819047	A	G	0.535	0.010	0.002	2.400E-08	0.00005	20.917	461053
Beef intake	rs79809011	8	10126532	A	G	0.029	-0.028	0.005	3.400E-08	0.00005	20.762	461053
Beef intake	rs11878917	19	42588999	A	G	0.110	0.015	0.003	4.600E-08	0.00004	20.313	461053
Fresh fruit intake	rs10249294	7	143723137	A	G	0.373	0.020	0.001	4.100E-54	0.00018	79.938	446462
Fresh fruit intake	rs1620977	1	72729142	G	A	0.731	-0.013	0.001	1.100E-21	0.00007	30.511	446462
Fresh fruit intake	rs994270	6	51187787	G	C	0.235	0.013	0.001	4.200E-20	0.00006	28.231	446462
Fresh fruit intake	rs10828266	10	22098701	G	A	0.716	0.012	0.001	8.100E-20	0.00006	27.781	446462
Fresh fruit intake	rs34162196	14	22038125	T	C	0.101	-0.018	0.002	4.000E-19	0.00006	26.639	446462
Fresh fruit intake	rs862227	16	73602926	G	A	0.458	-0.010	0.001	1.100E-16	0.00005	22.814	446462
Fresh fruit intake	rs2048522	18	44800515	T	A	0.435	0.010	0.001	1.800E-14	0.00004	20.068	446462
Fresh fruit intake	rs28479795	14	79943606	T	C	0.221	0.011	0.001	2.500E-14	0.00004	19.425	446462
Fresh fruit intake	rs73455661	18	57968685	G	A	0.279	0.010	0.001	4.100E-14	0.00004	19.048	446462
Fresh fruit intake	rs13072255	3	21038260	C	A	0.494	0.009	0.001	2.100E-13	0.00004	18.000	446462
Fresh fruit intake	rs2867113	2	651365	A	G	0.131	-0.014	0.002	1.500E-12	0.00004	19.522	446462
Fresh fruit intake	rs739320	19	49261368	C	T	0.606	-0.009	0.001	1.900E-12	0.00004	17.263	446462
Fresh fruit intake	rs10838724	11	47527052	T	G	0.368	0.009	0.001	2.100E-12	0.00004	16.863	446462
Fresh fruit intake	rs1964272	19	46190268	A	G	0.484	0.008	0.001	1.100E-11	0.00003	15.379	446462
Fresh fruit intake	rs2143081	6	50782834	A	G	0.540	0.008	0.001	1.300E-11	0.00003	15.358	446462
Fresh fruit intake	rs2790688	1	15399290	T	C	0.154	0.011	0.002	1.500E-11	0.00003	15.249	446462

Trait	SNP	chr	pos	EA	OA	eaf	beta	se	pval	R2	F	samplesize
			9									
Fresh fruit intake	rs11896330	2	60235568	A	G	0.633	-0.008	0.001	3.400E-11	0.00003	14.799	446462
Fresh fruit intake	rs4953150	2	45157336	T	C	0.344	-0.008	0.001	6.599E-11	0.00003	14.353	446462
Fresh fruit intake	rs62298288	4	59885755	T	C	0.433	0.008	0.001	1.400E-10	0.00003	13.745	446462
Fresh fruit intake	rs7554485	1	65945906	C	T	0.612	-0.008	0.001	1.700E-10	0.00003	13.611	446462
Fresh fruit intake	rs12420070	11	63985628	G	A	0.363	0.008	0.001	3.400E-10	0.00003	13.126	446462
Fresh fruit intake	rs12044599	1	204564714	G	A	0.210	0.009	0.002	3.700E-10	0.00003	13.151	446462
Fresh fruit intake	rs10192394	2	146298007	T	C	0.529	-0.008	0.001	4.500E-10	0.00003	13.058	446462
Fresh fruit intake	rs10064431	5	92950673	C	T	0.522	-0.008	0.001	6.000E-10	0.00003	12.780	446462
Fresh fruit intake	rs1375566	3	85642479	A	G	0.627	-0.008	0.001	6.100E-10	0.00003	12.810	446462
Fresh fruit intake	rs11085749	19	10961273	A	G	0.387	-0.008	0.001	7.100E-10	0.00003	12.670	446462
Fresh fruit intake	rs7982441	13	55924013	C	T	0.732	-0.008	0.001	9.800E-10	0.00003	12.401	446462
Fresh fruit intake	rs1051547	16	19279380	C	T	0.562	-0.008	0.001	1.100E-09	0.00003	12.616	446462
Fresh fruit intake	rs12885598	14	32071665	A	G	0.597	0.008	0.001	1.700E-09	0.00003	12.138	446462
Fresh fruit intake	rs7123429	11	8866808	G	C	0.555	0.007	0.001	1.900E-09	0.00003	12.094	446462
Fresh fruit intake	rs1866823	8	57436577	A	G	0.544	0.007	0.001	2.100E-09	0.00003	12.229	446462
Fresh fruit intake	rs149449	5	95902093	A	G	0.489	0.007	0.001	2.400E-09	0.00003	11.853	446462
Fresh fruit intake	rs817223	2	104094008	C	T	0.481	-0.007	0.001	2.800E-09	0.00003	11.779	446462
Fresh fruit intake	rs139042899	17	58443095	C	A	0.013	0.036	0.006	3.200E-09	0.00003	15.373	446462
Fresh fruit intake	rs1356292	3	185824903	T	C	0.808	0.009	0.002	3.500E-09	0.00003	11.682	446462
Fresh fruit intake	rs62051554	16	72826387	A	G	0.109	0.012	0.002	4.600E-09	0.00003	11.650	446462

Trait	SNP	chr	pos	EA	OA	eaf	beta	se	pval	R2	F	samplesize
Fresh fruit intake	rs11032362	11	33759092	A	G	0.091	0.012	0.002	5.300E-09	0.00003	11.342	446462
Fresh fruit intake	rs4744240	9	96218194	T	C	0.331	0.008	0.001	5.700E-09	0.00003	11.327	446462
Fresh fruit intake	rs17049185	2	58072660	T	G	0.268	0.008	0.001	7.300E-09	0.00003	11.321	446462
Fresh fruit intake	rs11248509	10	125134393	T	A	0.371	0.007	0.001	7.400E-09	0.00003	11.198	446462
Fresh fruit intake	rs12536253	7	127595077	C	G	0.249	-0.008	0.001	8.300E-09	0.00002	11.117	446462
Fresh fruit intake	rs559734	1	97304868	C	G	0.712	0.008	0.001	1.100E-08	0.00002	11.053	446462
Fresh fruit intake	rs4302893	9	1734863	A	G	0.334	0.007	0.001	1.300E-08	0.00002	10.846	446462
Fresh fruit intake	rs2093654	9	5780121	G	A	0.388	0.007	0.001	1.500E-08	0.00002	10.778	446462
Fresh fruit intake	rs9517948	13	100650708	T	C	0.451	0.007	0.001	1.700E-08	0.00002	10.690	446462
Fresh fruit intake	rs72974263	2	225447371	T	C	0.318	0.007	0.001	1.800E-08	0.00002	10.572	446462
Fresh fruit intake	rs6475724	9	23274223	T	C	0.727	0.008	0.001	1.900E-08	0.00002	10.560	446462
Fresh fruit intake	rs10271924	7	153495206	T	C	0.493	-0.007	0.001	2.000E-08	0.00002	11.083	446462
Fresh fruit intake	rs8095324	18	24131659	G	A	0.404	-0.007	0.001	2.700E-08	0.00002	10.374	446462
Fresh fruit intake	rs329277	7	35080931	G	T	0.486	0.007	0.001	2.800E-08	0.00002	10.397	446462
Fresh fruit intake	rs7818437	8	10209623	C	T	0.236	-0.008	0.001	3.000E-08	0.00002	10.425	446462
Fresh fruit intake	rs12780952	10	107577033	A	G	0.286	0.007	0.001	3.400E-08	0.00002	10.195	446462
Fresh fruit intake	rs7898861	10	65319678	C	T	0.486	-0.007	0.001	3.800E-08	0.00002	10.092	446462
Fresh fruit intake	rs78537042	21	33068937	A	C	0.087	-0.012	0.002	4.800E-08	0.00002	10.067	446462
Processed meat intake	rs838133	19	49259529	G	A	0.549	0.019	0.002	1.600E-18	0.00018	82.708	461981
Processed meat intake	rs4240672	8	10767917	A	G	0.494	0.017	0.002	3.000E-16	0.00015	67.703	461981

Trait	SNP	chr	pos	EA	OA	eaf	beta	se	pval	R2	F	samplesize
Processed meat intake	rs2029401	5	92891029	G	A	0.586	0.015	0.002	6.299E-12	0.00010	47.983	461981
Processed meat intake	rs7531118	1	72837239	C	T	0.531	-0.014	0.002	2.800E-11	0.00010	45.567	461981
Processed meat intake	rs6484504	11	31424823	C	T	0.725	0.015	0.002	4.400E-11	0.00010	44.144	461981
Processed meat intake	rs13091492	3	81891476	G	A	0.373	0.014	0.002	1.600E-10	0.00009	41.384	461981
Processed meat intake	rs9809856	3	18227421	G	A	0.476	0.013	0.002	2.500E-10	0.00009	40.699	461981
Processed meat intake	rs203319	22	41914593	T	C	0.205	-0.016	0.003	2.800E-10	0.00009	40.558	461981
Processed meat intake	rs11032380	11	33800533	T	A	0.333	-0.013	0.002	2.100E-09	0.00008	36.449	461981
Processed meat intake	rs77165542	2	430975	T	C	0.035	0.034	0.006	3.300E-09	0.00008	36.310	461981
Processed meat intake	rs1422192	5	87959023	A	G	0.158	0.017	0.003	3.400E-09	0.00008	35.407	461981
Processed meat intake	rs10454812	5	52800358	C	A	0.103	-0.020	0.003	6.700E-09	0.00007	34.021	461981
Processed meat intake	rs6961970	7	113901132	A	C	0.245	-0.014	0.002	9.500E-09	0.00007	33.541	461981
Processed meat intake	rs11894162	2	107197420	T	C	0.547	0.012	0.002	1.100E-08	0.00007	33.234	461981
Processed meat intake	rs34241936	17	74065908	G	A	0.037	0.033	0.006	1.100E-08	0.00008	35.841	461981
Processed meat intake	rs6010651	20	62418243	C	A	0.379	-0.012	0.002	1.100E-08	0.00007	33.486	461981
Processed meat intake	rs4778053	15	93424341	G	C	0.844	0.016	0.003	1.300E-08	0.00007	33.022	461981
Processed meat intake	rs6765179	3	25276416	A	G	0.310	-0.013	0.002	1.800E-08	0.00007	32.180	461981
Processed meat intake	rs6786550	3	62560523	C	T	0.635	0.012	0.002	2.100E-08	0.00007	31.807	461981
Processed meat intake	rs11887120	2	25485735	T	C	0.398	0.012	0.002	3.100E-08	0.00007	31.671	461981
Processed meat intake	rs3762621	2	173306140	T	C	0.183	-0.015	0.003	3.600E-08	0.00007	31.100	461981
Processed meat intake	rs4077924	2	181991389	C	T	0.702	0.012	0.002	4.500E-08	0.00007	30.203	461981
Processed	rs8096	18	3480	C	T	0.193	-0.015	0.003	4.700	0.0000	30.602	4619

Trait	SNP	chr	pos	EA	OA	eaf	beta	se	pval	R2	F	samplesize
meat intake	167		0257						E-08	7		81
Cooked vegetable intake	rs12629972	3	25121444	C	T	0.588	0.012	0.002	1.200E-13	0.00007	30.260	448651
Cooked vegetable intake	rs838133	19	49259529	G	A	0.550	0.012	0.002	4.500E-13	0.00007	30.344	448651
Cooked vegetable intake	rs10156602	9	96345328	G	A	0.361	0.011	0.002	1.800E-11	0.00006	25.087	448651
Cooked vegetable intake	rs28711392	11	13349559	C	T	0.367	-0.011	0.002	4.600E-11	0.00005	24.077	448651
Cooked vegetable intake	rs4851029	2	104159785	G	T	0.527	0.010	0.002	7.800E-11	0.00005	23.155	448651
Cooked vegetable intake	rs1421085	16	53800954	C	T	0.403	0.010	0.002	8.300E-11	0.00005	23.053	448651
Cooked vegetable intake	rs2052063	10	118040352	T	C	0.516	-0.009	0.002	1.600E-09	0.00004	20.045	448651
Cooked vegetable intake	rs2844672	6	31005139	A	G	0.624	-0.010	0.002	2.100E-09	0.00004	19.571	448651
Cooked vegetable intake	rs2102738	2	172525884	C	A	0.172	-0.012	0.002	5.300E-09	0.00004	18.925	448651
Cooked vegetable intake	rs2252508	1	153913770	G	A	0.480	0.009	0.002	5.700E-09	0.00004	18.557	448651
Cooked vegetable intake	rs10161952	13	59474383	C	A	0.313	-0.010	0.002	1.300E-08	0.00004	17.700	448651
Cooked vegetable intake	rs12550717	8	10266706	A	G	0.372	0.009	0.002	1.400E-08	0.00004	17.703	448651
Cooked vegetable intake	rs11138705	9	83121545	C	G	0.757	0.010	0.002	1.400E-08	0.00004	17.746	448651
Cooked vegetable intake	rs349062	11	84788804	C	G	0.393	-0.009	0.002	2.500E-08	0.00004	16.967	448651
Cooked vegetable intake	rs1816263	5	141217079	C	T	0.280	0.010	0.002	3.700E-08	0.00004	16.614	448651
Cooked	rs3415	22	4897	T	C	0.227	0.011	0.002	3.900	0.0000	17.574	4486

Trait	SNP	chr	pos	EA	OA	eaf	beta	se	pval	R2	F	samplesize
vegetable intake	5012		7215						E-08	4		51
Cooked vegetable intake	rs28450747	4	42137653	A	G	0.233	-0.010	0.002	4.300E-08	0.00004	16.534	448651
Pork intake	rs1355171	9	15681694	A	C	0.489	-0.011	0.001	1.000E-13	0.00006	27.743	460162
Pork intake	rs838133	19	49259529	G	A	0.549	0.011	0.002	8.999E-13	0.00006	27.043	460162
Pork intake	rs9379832	6	26186200	G	A	0.255	-0.011	0.002	1.800E-11	0.00005	23.036	460162
Pork intake	rs12721051	19	45422160	G	C	0.188	-0.012	0.002	5.600E-11	0.00005	21.511	460162
Pork intake	rs10972033	9	34269732	T	G	0.456	0.009	0.001	1.300E-09	0.00004	18.395	460162
Pork intake	rs3964074	16	74166991	C	T	0.547	-0.009	0.001	1.600E-09	0.00004	18.272	460162
Pork intake	rs254152	5	115641827	G	C	0.235	-0.010	0.002	2.200E-09	0.00004	17.986	460162
Pork intake	rs4146837	15	93446869	T	C	0.456	0.009	0.001	4.000E-09	0.00004	17.646	460162
Pork intake	rs34161520	10	125127645	G	C	0.160	0.012	0.002	9.600E-09	0.00004	16.653	460162
Pork intake	rs9973426	2	155751852	G	A	0.177	0.011	0.002	1.000E-08	0.00004	16.444	460162
Pork intake	rs11211124	1	45962409	C	T	0.231	-0.010	0.002	1.400E-08	0.00004	16.175	460162
Pork intake	rs36124222	18	74196202	C	T	0.433	0.008	0.002	2.100E-08	0.00003	15.981	460162
Pork intake	rs2387807	12	38446861	T	C	0.078	-0.015	0.003	4.100E-08	0.00003	15.042	460162
Pork intake	rs7641973	3	81910489	A	G	0.353	0.008	0.002	4.200E-08	0.00003	15.002	460162
Poultry intake	rs7046351	9	15664421	A	T	0.510	0.011	0.002	1.100E-09	0.00006	28.317	461900
Poultry intake	rs7829800	8	144258705	G	A	0.671	0.011	0.002	3.700E-09	0.00006	26.785	461900
Poultry intake	rs2565017	18	21070280	A	G	0.373	0.011	0.002	5.900E-09	0.00006	25.905	461900
Poultry intake	rs9923768	16	6163838	A	G	0.599	0.011	0.002	1.600E-08	0.00005	24.532	461900

Trait	SNP	chr	pos	EA	OA	eaf	beta	se	pval	R2	F	samplesize
Poultry intake	rs1051730	15	78894339	A	G	0.331	-0.011	0.002	1.700E-08	0.00005	24.212	461900
Poultry intake	rs9997448	4	140870515	T	C	0.369	-0.010	0.002	2.700E-08	0.00005	23.562	461900
Poultry intake	rs2965200	19	19476365	A	G	0.640	-0.010	0.002	4.200E-08	0.00005	23.109	461900
Poultry intake	rs2426440	20	50999841	G	A	0.733	0.011	0.002	4.700E-08	0.00005	22.774	461900
Lamb/mutton intake	rs276453	9	15573753	C	A	0.488	-0.014	0.001	2.900E-22	0.00010	46.599	460006
Lamb/mutton intake	rs429358	19	45411941	C	T	0.154	-0.018	0.002	2.700E-19	0.00009	39.728	460006
Lamb/mutton intake	rs3105087	13	55948790	C	T	0.733	-0.012	0.002	1.800E-12	0.00005	24.363	460006
Lamb/mutton intake	rs4489752	11	126590934	T	G	0.836	0.014	0.002	2.800E-12	0.00005	24.060	460006
Lamb/mutton intake	rs2678900	2	58177683	G	T	0.428	0.010	0.001	9.899E-12	0.00005	22.905	460006
Lamb/mutton intake	rs11090045	22	41753603	A	G	0.307	-0.011	0.002	3.000E-11	0.00005	22.316	460006
Lamb/mutton intake	rs55813438	16	5694999	A	G	0.763	-0.011	0.002	4.700E-11	0.00005	21.680	460006
Lamb/mutton intake	rs2726033	16	28338039	G	A	0.422	-0.009	0.001	1.500E-10	0.00004	20.214	460006
Lamb/mutton intake	rs62106258	2	417167	C	T	0.049	0.022	0.003	2.000E-10	0.00004	19.889	460006
Lamb/mutton intake	rs7447465	5	164566362	C	T	0.619	0.010	0.002	2.000E-10	0.00004	19.915	460006
Lamb/mutton intake	rs136548	22	27253353	T	C	0.377	0.010	0.002	2.900E-10	0.00004	19.629	460006
Lamb/mutton intake	rs660880	1	6866978	A	G	0.513	-0.009	0.001	6.800E-10	0.00004	18.753	460006
Lamb/mutton intake	rs7550173	1	72944617	T	A	0.610	-0.009	0.002	1.300E-09	0.00004	18.216	460006
Lamb/mutton intake	rs35797675	7	72878044	G	T	0.216	-0.011	0.002	1.400E-09	0.00004	18.337	460006
Lamb/mutton intake	rs139237013	2	56662691	A	G	0.058	0.019	0.003	1.800E-09	0.00004	17.857	460006
Lamb/mutton intake	rs11743441	5	88065637	T	G	0.574	-0.009	0.001	2.700E-09	0.00004	17.585	460006
Lamb/mutton intake	rs12634740	3	17567145	G	T	0.252	-0.010	0.002	2.800E-09	0.00004	17.649	460006

Trait	SNP	chr	pos	EA	OA	eaf	beta	se	pval	R2	F	samplesize
			0									
Lamb/mutton intake	rs673696	11	31097262	T	C	0.081	0.016	0.003	3.700E-09	0.00004	17.141	460006
Lamb/mutton intake	rs62398404	6	37669616	T	C	0.127	0.013	0.002	4.000E-09	0.00004	17.039	460006
Lamb/mutton intake	rs4272399	8	4836291	A	C	0.321	-0.009	0.002	4.500E-09	0.00004	17.143	460006
Lamb/mutton intake	rs1556147	9	131936055	T	A	0.672	0.009	0.002	5.300E-09	0.00004	16.808	460006
Lamb/mutton intake	rs2140714	3	83786985	G	C	0.558	-0.009	0.001	7.700E-09	0.00004	16.492	460006
Lamb/mutton intake	rs6829572	4	93984155	A	G	0.457	0.008	0.001	1.200E-08	0.00004	16.112	460006
Lamb/mutton intake	rs994270	6	51187787	G	C	0.235	0.010	0.002	1.400E-08	0.00003	15.944	460006
Lamb/mutton intake	rs16891982	5	33951693	G	C	0.972	-0.024	0.004	2.700E-08	0.00003	14.674	460006
Lamb/mutton intake	rs2222760	2	151345113	A	G	0.281	-0.009	0.002	2.800E-08	0.00003	15.365	460006
Lamb/mutton intake	rs56394517	1	243582753	G	A	0.096	-0.014	0.002	3.200E-08	0.00003	15.128	460006
Lamb/mutton intake	rs1958801	14	98872110	G	A	0.288	-0.009	0.002	3.200E-08	0.00003	15.091	460006
Lamb/mutton intake	rs3964074	16	74166991	C	T	0.547	-0.008	0.001	3.200E-08	0.00003	15.110	460006
Lamb/mutton intake	rs6581296	12	60473007	G	C	0.795	0.010	0.002	4.000E-08	0.00003	15.070	460006
Lamb/mutton intake	rs17270057	19	42670527	C	T	0.113	0.013	0.002	4.300E-08	0.00003	14.804	460006
Lamb/mutton intake	rs2926119	16	64293179	A	C	0.569	0.008	0.001	4.400E-08	0.00003	14.841	460006
Dried fruit intake	rs10740991	10	22058137	C	G	0.718	0.017	0.002	2.000E-19	0.00011	47.901	421764
Dried fruit intake	rs429358	19	45411941	C	T	0.154	0.020	0.002	6.700E-18	0.00010	43.768	421764
Dried fruit intake	rs11772627	7	2109821	C	G	0.182	0.018	0.002	3.000E-17	0.00010	42.219	421764
Dried fruit intake	rs3101339	1	72748669	C	A	0.603	0.014	0.002	6.200E-17	0.00010	41.053	421764
Dried fruit intake	rs10896126	11	66292908	G	A	0.304	-0.015	0.002	1.600E-16	0.00010	40.178	421764
Dried fruit	rs3416	14	2203	T	C	0.101	-0.022	0.003	7.101	0.0000	38.307	4217

Trait	SNP	chr	pos	EA	OA	eaf	beta	se	pval	R2	F	samplesize
intake	2196		8125						E-16	9		64
Dried fruit intake	rs746868	6	31540429	G	C	0.615	-0.013	0.002	5.200E-14	0.00008	33.278	421764
Dried fruit intake	rs4269101	3	18763543	G	T	0.719	-0.014	0.002	1.100E-13	0.00008	32.508	421764
Dried fruit intake	rs9385269	6	98547979	T	C	0.525	0.012	0.002	7.199E-13	0.00007	30.637	421764
Dried fruit intake	rs4800488	18	21117571	A	C	0.490	0.012	0.002	7.700E-13	0.00007	30.274	421764
Dried fruit intake	rs4149513	2	101022726	A	G	0.494	0.012	0.002	2.200E-12	0.00007	29.013	421764
Dried fruit intake	rs3764002	12	108618630	T	C	0.261	0.013	0.002	5.100E-12	0.00007	28.043	421764
Dried fruit intake	rs2328887	6	25430149	C	T	0.899	0.019	0.003	8.800E-12	0.00006	27.388	421764
Dried fruit intake	rs11811826	1	204603861	A	T	0.224	0.013	0.002	4.400E-11	0.00006	25.637	421764
Dried fruit intake	rs7808471	7	132716502	C	T	0.322	-0.012	0.002	1.100E-10	0.00006	24.515	421764
Dried fruit intake	rs11037497	11	43622423	C	G	0.446	0.010	0.002	5.700E-10	0.00005	22.718	421764
Dried fruit intake	rs7599488	2	60718347	T	C	0.426	-0.010	0.002	6.700E-10	0.00005	22.382	421764
Dried fruit intake	rs75641275	1	98327133	C	A	0.143	-0.014	0.002	2.900E-09	0.00005	20.777	421764
Dried fruit intake	rs1622515	11	95523433	G	A	0.485	0.010	0.002	2.900E-09	0.00005	20.722	421764
Dried fruit intake	rs62084586	17	56419228	C	T	0.166	0.013	0.002	3.200E-09	0.00005	20.926	421764
Dried fruit intake	rs10026792	4	2862190	A	G	0.290	0.011	0.002	3.900E-09	0.00005	20.451	421764
Dried fruit intake	rs2533273	7	153485282	A	C	0.485	-0.010	0.002	3.900E-09	0.00005	20.552	421764
Dried fruit intake	rs7829800	8	144258705	G	A	0.671	-0.010	0.002	5.100E-09	0.00005	20.320	421764
Dried fruit intake	rs17175518	18	57850583	A	C	0.233	0.011	0.002	5.900E-09	0.00005	19.911	421764
Dried fruit intake	rs1582322	16	52105988	G	A	0.605	0.010	0.002	6.800E-09	0.00005	19.935	421764

Trait	SNP	chr	pos	EA	OA	eaf	beta	se	pval	R2	F	samplesize
Dried fruit intake	rs11720884	3	43941406	G	A	0.250	0.011	0.002	7.600E-09	0.00005	19.776	421764
Dried fruit intake	rs57499472	3	147239337	C	T	0.404	0.010	0.002	8.100E-09	0.00005	19.959	421764
Dried fruit intake	rs72720396	1	91191582	G	A	0.229	0.011	0.002	8.700E-09	0.00005	19.456	421764
Dried fruit intake	rs7582086	2	60231826	T	G	0.468	-0.010	0.002	8.800E-09	0.00005	19.471	421764
Dried fruit intake	rs1797235	15	47821612	C	G	0.375	-0.010	0.002	8.900E-09	0.00005	19.845	421764
Dried fruit intake	rs7916868	10	64988931	T	A	0.503	0.010	0.002	9.100E-09	0.00005	19.465	421764
Dried fruit intake	rs261809	1	241054465	G	A	0.541	-0.010	0.002	9.800E-09	0.00005	19.422	421764
Dried fruit intake	rs11586016	1	44031793	C	G	0.371	0.010	0.002	1.100E-08	0.00005	19.209	421764
Dried fruit intake	rs893856	10	126723567	A	G	0.149	-0.013	0.002	1.300E-08	0.00005	19.093	421764
Dried fruit intake	rs8081370	17	1373612	T	C	0.910	-0.017	0.003	1.400E-08	0.00005	19.146	421764
Dried fruit intake	rs1648404	4	37175523	T	C	0.476	0.009	0.002	1.800E-08	0.00004	18.655	421764
Dried fruit intake	rs4140799	14	72170969	A	G	0.532	0.009	0.002	1.800E-08	0.00004	18.783	421764
Dried fruit intake	rs17184707	2	166183577	T	C	0.213	-0.011	0.002	2.100E-08	0.00004	18.488	421764
Dried fruit intake	rs10129747	14	77433198	G	A	0.530	0.009	0.002	2.600E-08	0.00004	18.405	421764
Dried fruit intake	rs12137234	1	72270797	T	C	0.304	0.010	0.002	2.800E-08	0.00004	18.580	421764
Dried fruit intake	rs862227	16	73602926	G	A	0.458	-0.009	0.002	4.300E-08	0.00004	17.590	421764
Dried fruit intake	rs11632215	15	45319982	C	A	0.120	-0.014	0.003	4.400E-08	0.00004	17.842	421764
Dried fruit intake	rs11152349	18	60233646	A	G	0.303	0.010	0.002	4.900E-08	0.00004	17.499	421764
Non-oily fish intake	rs838133	19	49259529	G	A	0.549	0.016	0.002	4.700E-22	0.00013	59.775	460880
Non-oily fish intake	rs56094641	16	53806453	G	A	0.405	0.013	0.002	2.500E-14	0.00008	35.186	460880
Non-oily fish	rs4318	6	3276	T	C	0.177	-0.015	0.002	1.300	0.0000	30.398	4608

Trait	SNP	chr	pos	EA	OA	eaf	beta	se	pval	R2	F	samplesize
intake	925		1506						E-12	7		80
Non-oily fish intake	rs35287743	12	110057250	T	G	0.116	-0.018	0.003	3.600E-12	0.00006	29.711	460880
Non-oily fish intake	rs3799077	6	69996217	G	T	0.310	-0.011	0.002	1.000E-09	0.00005	22.720	460880
Non-oily fish intake	rs16822430	2	144147475	C	T	0.233	0.012	0.002	1.400E-09	0.00005	22.313	460880
Non-oily fish intake	rs11680516	2	208205711	C	T	0.202	0.012	0.002	1.400E-09	0.00005	22.455	460880
Non-oily fish intake	rs6957745	7	73056750	C	T	0.203	-0.012	0.002	1.800E-09	0.00005	22.120	460880
Non-oily fish intake	rs1260326	2	27730940	C	T	0.604	-0.010	0.002	7.900E-09	0.00004	20.119	460880
Non-oily fish intake	rs7148387	14	29775132	G	A	0.591	-0.009	0.002	1.700E-08	0.00004	19.302	460880
Non-oily fish intake	rs17317920	7	25517103	G	A	0.479238	0.00906029	0.0016312	2.80001E-08	4.09737E-05	18.88463081	460880

Abbreviations: SNP: single nucleotide polymorphism; EA: effect Allele; OA: other Allele; EAF: effect allele frequency; SE: standard error.

Table.S4 MR analysis results

id.exposure	id.outcome	nSNP	P_IVW	b_IVW	Hetero_P_MR.Egger	Hetero_P_IVW	Pleiotropy_P	or	or_lci95	or_uci95	MR.PRESSO_P
ukb-b-2862	id:ebi-a-GCST006909	15	0.35	-0.63	0.11	0.13	0.56	0.53	0.15	1.97	0.01
ukb-b-14179	id:ebi-a-GCST006909	31	0.94	-0.03	0.45	0.50	0.78	0.97	0.39	2.38	0.532
ukb-b-5640	id:ebi-a-GCST006909	14	0.841	-0.206	0.005	0.007	0.576	0.814	0.109	6.084	0.007
ukb-b-8006	id:ebi-a-GCST006909	7	0.345	1.120	0.134	0.087	0.268	3.065	0.300	31.313	0.018
ukb-b-2209	id:ebi-a-GCST006909	60	0.768	-0.080	0.040	0.048	0.822	0.923	0.540	1.576	0.040
ukb-b-17627	id:ebi-a-GCST006909	11	0.344	-0.628	0.796	0.821	0.493	0.533	0.145	1.958	0.803
ukb-b-16576	id:ebi-a-GCST006909	41	0.005	-0.966	0.376	0.394	0.453	0.381	0.195	0.743	0.176
ukb-b-3881	id:ebi-a-GCST006909	52	0.825	0.111	0.004	0.002	0.078	1.117	0.418	2.985	0.001
ukb-b-1996	id:ebi-a-GCST006909	18	0.746	0.295	0.008	0.008	0.440	1.343	0.226	7.984	0.018
ukb-b-8089	id:ebi-a-GCST006909	17	0.713	-0.219	0.799	0.817	0.468	0.803	0.249	2.585	0.823
ukb-b-5237	id:ebi-a-GCST006909	38	0.168	-0.406	0.261	0.295	0.745	0.666	0.374	1.187	0.297
ukb-b-6324	id:ebi-a-GCST006909	23	0.704	-0.234	0.000	0.000	0.253	0.791	0.237	2.642	<0.001
ukb-b-2862	id:ebi-a-GCST90014122	11	0.866	0.122	0.076	0.112	0.972	1.130	0.273	4.680	<0.001
ukb-b-14179	id:ebi-a-GCST90014122	26	0.550	-0.266	0.575	0.627	0.780	0.766	0.320	1.833	0.413

id.exposure	id.outcome	nSNP	P_IVW	b_IVW	Hetero_P_MR.Egger	Hetero_P_IVW	Pleiotropy_P	or	or_lci95	or_uci95	MR.PRESSO_P
ukb-b-5640	id:ebi-a-GCST90014122	11	0.440	-0.926	0.002	0.001	0.280	0.396	0.038	4.144	0.004
ukb-b-8006	id:ebi-a-GCST90014122	7	0.110	1.229	0.589	0.509	0.269	3.418	0.758	15.424	0.624
ukb-b-2209	id:ebi-a-GCST90014122	52	0.300	-0.247	0.307	0.329	0.559	0.781	0.489	1.246	0.467
ukb-b-17627	id:ebi-a-GCST90014122	11	0.020	-1.363	0.390	0.474	0.769	0.256	0.081	0.807	0.452
ukb-b-16576	id:ebi-a-GCST90014122	35	0.003	-1.141	0.073	0.089	0.775	0.320	0.149	0.686	0.050
ukb-b-3881	id:ebi-a-GCST90014122	46	0.771	0.136	0.010	0.010	0.407	1.145	0.459	2.859	<0.001
ukb-b-1996	id:ebi-a-GCST90014122	11	0.820	-0.186	0.283	0.282	0.356	0.830	0.168	4.108	0.174
ukb-b-8089	id:ebi-a-GCST90014122	15	0.095	-0.960	0.394	0.423	0.447	0.383	0.124	1.184	0.279
ukb-b-5237	id:ebi-a-GCST90014122	32	0.493	-0.178	0.547	0.598	0.908	0.837	0.504	1.391	0.563
ukb-b-6324	id:ebi-a-GCST90014122	21	0.621	-0.286	0.000	0.000	0.294	0.752	0.243	2.329	<0.001
ukb-b-2862	id:ebi-a-GCST90018870	14	0.929	0.081	0.087	0.113	0.685	1.085	0.182	6.462	0.147
ukb-b-14179	id:ebi-a-GCST	30	0.827	-0.158	0.043	0.005	0.015	0.854	0.208	3.509	0.003

id.exposure	id.outcome	nSNP	P_IVW	b_IVW	Hetero_P_MR.Egger	Hetero_P_IVW	Pleiotropy_P	or	or_lci95	or_uci95	MR.PRESSO_P
	90018870										
ukb-b-5640	id:ebi-a-GCST90018870	14	0.041	-1.777	0.219	0.182	0.241	0.169	0.031	0.930	0.182
ukb-b-8006	id:ebi-a-GCST90018870	7	0.090	1.972	0.369	0.194	0.143	7.183	0.733	70.396	0.141
ukb-b-2209	id:ebi-a-GCST90018870	61	0.278	-0.295	0.578	0.613	0.830	0.745	0.437	1.269	0.634
ukb-b-17627	id:ebi-a-GCST90018870	11	0.850	-0.193	0.044	0.066	0.842	0.825	0.112	6.050	0.071
ukb-b-16576	id:ebi-a-GCST90018870	41	0.634	0.197	0.193	0.194	0.347	1.217	0.541	2.737	0.141
ukb-b-3881	id:ebi-a-GCST90018870	52	0.176	-0.627	0.298	0.321	0.567	0.534	0.216	1.325	0.247
ukb-b-1996	id:ebi-a-GCST90018870	18	0.627	0.375	0.893	0.920	0.741	1.455	0.321	6.590	0.947
ukb-b-8089	id:ebi-a-GCST90018870	17	0.062	1.249	0.507	0.501	0.315	3.486	0.941	12.913	0.534
ukb-b-5237	id:ebi-a-GCST90018870	38	0.916	-0.035	0.304	0.344	0.835	0.966	0.506	1.842	0.375
ukb-b-6324	id:ebi-a-GCST90018870	23	0.122	0.685	0.493	0.555	0.984	1.983	0.833	4.720	0.577
ukb-b-16576	id:ebi-a-GCST90018923	41	0.635	-0.193	0.440	0.485	0.919	0.825	0.372	1.828	0.516

id.exposure	id.outcome	nSNP	P_IVW	b_IVW	Hetero_P_MR.Egger	Hetero_P_IVW	Pleiotropy_P	or	or_lci95	or_uci95	MR.PRESSO_P
ukb-b-2209	id:ebi-a-GCST90018923	61	0.681	0.119	0.701	0.728	0.703	1.127	0.637	1.993	0.716
ukb-b-6324	id:ebi-a-GCST90018923	23	0.724	0.167	0.525	0.586	0.978	1.182	0.468	2.986	0.609
ukb-b-1996	id:ebi-a-GCST90018923	18	0.768	-0.292	0.277	0.107	0.044	0.747	0.108	5.170	0.195
ukb-b-17627	id:ebi-a-GCST90018923	11	0.649	0.442	0.164	0.174	0.427	1.556	0.231	10.478	0.171
ukb-b-3881	id:ebi-a-GCST90018923	52	0.861	0.088	0.295	0.264	0.179	1.092	0.407	2.932	0.283
ukb-b-2862	id:ebi-a-GCST90018923	14	0.084	1.372	0.473	0.553	0.856	3.942	0.830	18.720	0.329
ukb-b-5640	id:ebi-a-GCST90018923	14	0.758	-0.287	0.138	0.185	0.921	0.750	0.121	4.645	0.187
ukb-b-8089	id:ebi-a-GCST90018923	17	0.837	-0.148	0.866	0.886	0.534	0.863	0.212	3.509	0.903
ukb-b-8006	id:ebi-a-GCST90018923	7	0.947	-0.069	0.760	0.856	0.944	0.933	0.122	7.149	0.857
ukb-b-14179	id:ebi-a-GCST90018923	31	0.304	0.637	0.179	0.195	0.499	1.892	0.561	6.375	0.161
ukb-b-5237	id:ebi-a-GCST90018923	38	0.470	0.245	0.604	0.648	0.881	1.277	0.658	2.482	0.740
ukb-b-2862	id:ebi-a-GCST	15	0.354	0.006	0.083	0.113	0.819	1.006	0.993	1.020	0.064

id.exposure	id.outcome	nSNP	P_IVW	b_IVW	Hetero_P_MR.Egger	Hetero_P_IVW	Pleiotropy_P	or	or_lci95	or_uci95	MR.PRESSO_P
	90038613										
ukb-b-14179	id:ebi-a-GCST90038613	31	0.938	0.000	0.027	0.036	0.965	1.000	0.988	1.011	0.041
ukb-b-5640	id:ebi-a-GCST90038613	14	0.450	0.008	0.004	0.003	0.393	1.008	0.987	1.030	0.003
ukb-b-8006	id:ebi-a-GCST90038613	7	0.720	0.005	0.006	0.011	0.728	1.005	0.977	1.035	0.008
ukb-b-2209	id:ebi-a-GCST90038613	61	0.016	-0.006	0.107	0.124	0.935	0.994	0.988	0.999	0.059
ukb-b-17627	id:ebi-a-GCST90038613	11	0.303	-0.008	0.558	0.198	0.041	0.992	0.977	1.007	0.166
ukb-b-16576	id:ebi-a-GCST90038613	41	0.013	-0.009	0.168	0.190	0.641	0.991	0.983	0.998	0.151
ukb-b-3881	id:ebi-a-GCST90038613	52	0.312	-0.004	0.420	0.436	0.444	0.996	0.988	1.004	0.439
ukb-b-1996	id:ebi-a-GCST90038613	18	0.269	-0.007	0.707	0.708	0.360	0.993	0.981	1.005	0.694
ukb-b-8089	id:ebi-a-GCST90038613	17	0.576	-0.004	0.224	0.118	0.091	0.996	0.982	1.010	0.112
ukb-b-5237	id:ebi-a-GCST90038613	38	0.963	0.000	0.116	0.129	0.547	1.000	0.994	1.006	0.138
ukb-b-6324	id:ebi-a-GCST90038613	23	0.142	0.007	0.098	0.034	0.055	1.007	0.998	1.017	0.023

id.exposure	id.outcome	nSNP	P_IVW	b_IVW	Hetero_P_MR.Egger	Hetero_P_IVW	Pleiotropy_P	or	or_lci95	or_uci95	MR.PRESSO_P
ukb-b-2862	id:finn-b-I9_STR_EXH	14	0.810	-0.094	0.293	0.365	0.949	0.910	0.424	1.955	0.354
ukb-b-14179	id:finn-b-I9_STR_EXH	31	0.597	-0.159	0.907	0.928	0.951	0.853	0.472	1.541	0.931
ukb-b-5640	id:finn-b-I9_STR_EXH	13	0.731	0.258	0.001	0.001	0.576	1.294	0.297	5.637	0.003
ukb-b-8006	id:finn-b-I9_STR_EXH	7	0.106	0.921	0.852	0.901	0.664	2.511	0.822	7.671	0.476
ukb-b-2209	id:finn-b-I9_STR_EXH	61	0.882	-0.027	0.045	0.027	0.081	0.973	0.681	1.391	0.023
ukb-b-17627	id:finn-b-I9_STR_EXH	11	0.067	-1.125	0.022	0.025	0.487	0.325	0.097	1.084	0.024
ukb-b-16576	id:finn-b-I9_STR_EXH	41	0.034	-0.455	0.480	0.517	0.675	0.634	0.417	0.965	0.598
ukb-b-3881	id:finn-b-I9_STR_EXH	52	0.969	-0.011	0.059	0.069	0.703	0.989	0.559	1.747	0.043
ukb-b-1996	id:finn-b-I9_STR_EXH	18	0.930	-0.040	0.278	0.326	0.675	0.961	0.392	2.354	0.214
ukb-b-8089	id:finn-b-I9_STR_EXH	17	0.907	0.056	0.153	0.081	0.112	1.058	0.415	2.697	0.064
ukb-b-5237	id:finn-b-I9_STR_EXH	38	0.997	0.001	0.119	0.082	0.116	1.001	0.668	1.499	0.125
ukb-b-6324	id:finn-b-I9_STR_EXH	23	0.91957578	-0.031740857	0.027033549	0.031352771	0.525416342	0.968757596	0.523144988	1.793941069	0.023

Abbreviations: P_IVW: Inverse Variance Weighted method P-value; b_IVW: Inverse Variance Weighted method beta coefficient; Hetero_P_MR.Egger: MR-Egger regression-based heterogeneity test P-value; Hetero_P_IVW: IVW-based heterogeneity test P-value; Pleiotropy_P: Horizontal pleiotropy test P-value; OR: Odds Ratio; OR_lci95: OR 95% confidence interval lower limit; OR_uci95: OR 95% confidence interval upper limit; MR.PRESSO_P: MR-PRESSO (Mendelian Randomization Pleiotropy RESidual Sum and Outlier) global test P-value.

Table.S5 Reverse MR analysis

outcome	exposure	method	nsnp	b	se	pval
Oily fish intake	Stroke	MR Egger	23	-0.016	0.765	0.984
Oily fish intake	Stroke	Weighted median	23	-0.636	0.490	0.194
Oily fish intake	Stroke	Inverse variance weighted	23	-0.856	0.356	0.016
Oily fish intake	Stroke	Simple mode	23	-0.608	0.967	0.536
Oily fish intake	Stroke	Weighted mode	23	-0.627	0.933	0.509
Pork intake	Intracerebral hemorrhage	MR Egger	21	0.001	0.004	0.776
Pork intake	Intracerebral hemorrhage	Weighted median	21	0.002	0.003	0.500
Pork intake	Intracerebral hemorrhage	Inverse variance weighted	21	0.000	0.002	0.870
Pork intake	Intracerebral hemorrhage	Simple mode	21	0.002	0.004	0.688
Pork intake	Intracerebral hemorrhage	Weighted mode	21	0.002	0.004	0.596
Non-oily fish intake	Lacunar stroke	MR Egger	42	-0.009	0.007	0.199
Non-oily fish intake	Lacunar stroke	Weighted median	42	-0.003	0.003	0.325
Non-oily fish intake	Lacunar stroke	Inverse variance weighted	42	-0.003	0.003	0.277
Non-oily fish intake	Lacunar stroke	Simple mode	42	-0.006	0.008	0.475
Non-oily fish intake	Lacunar stroke	Weighted mode	42	-0.005	0.008	0.493
Dried fruit intake	Lacunar stroke	MR Egger	42	-0.008	0.008	0.302
Dried fruit intake	Lacunar stroke	Weighted median	42	-0.008	0.004	0.026
Dried fruit intake	Lacunar stroke	Inverse variance weighted	42	-0.007	0.003	0.016
Dried fruit intake	Lacunar stroke	Simple mode	42	-0.008	0.008	0.288
Dried fruit intake	Lacunar stroke	Weighted mode	42	-0.009	0.008	0.263
Dried fruit intake	Ischemic stroke (small-vessel)	MR Egger	61	0.001	0.004	0.804
Dried fruit intake	Ischemic stroke (small-vessel)	Weighted median	61	-0.001	0.003	0.597
Dried fruit intake	Ischemic stroke (small-vessel)	Inverse variance weighted	61	-0.001	0.002	0.740
Dried fruit intake	Ischemic stroke (small-vessel)	Simple mode	61	-0.002	0.007	0.735
Dried fruit	Ischemic stroke (small-vessel)	Weighted mode	61	-0.002	0.006	0.725

outcome	exposure	method	nsnp	b	se	pval
intake						
Dried fruit intake	Ischaemic Stroke, excluding all haemorrhages	MR Egger	23	0.004	0.017	0.830
Dried fruit intake	Ischaemic Stroke, excluding all haemorrhages	Weighted median	23	0.001	0.007	0.869
Dried fruit intake	Ischaemic Stroke, excluding all haemorrhages	Inverse variance weighted	23	0.002	0.006	0.752
Dried fruit intake	Ischaemic Stroke, excluding all haemorrhages	Simple mode	23	0.001	0.011	0.912
Dried fruit intake	Ischaemic Stroke, excluding all haemorrhages	Weighted mode	23	0.000	0.010	0.972
Dried fruit intake	Stroke	MR Egger	23	0.086	0.668	0.899
Dried fruit intake	Stroke	Weighted median	23	-0.023	0.422	0.957
Dried fruit intake	Stroke	Inverse variance weighted	23	-0.454	0.311	0.144
Dried fruit intake	Stroke	Simple mode	23	0.256	0.837	0.763
Dried fruit intake	Stroke	Weighted mode	23	0.307	0.749	0.686
