# Supplementary material for: Exploring causal links between multifaceted dietary exposures and stroke subtypes: Results from a two-sample Mendelian randomization analysis
Source: Medicine (Baltimore). 2026 Apr 17;105(16):e48375. doi: 10.1097/MD.0000000000048375 (PMC13095255; doi:10.1097/MD.0000000000048375)
Supplement: Supplementary file 2 [file medi-105-e48375-s002.pdf]

Fig.S1 Scatter Plots of MR Analyses

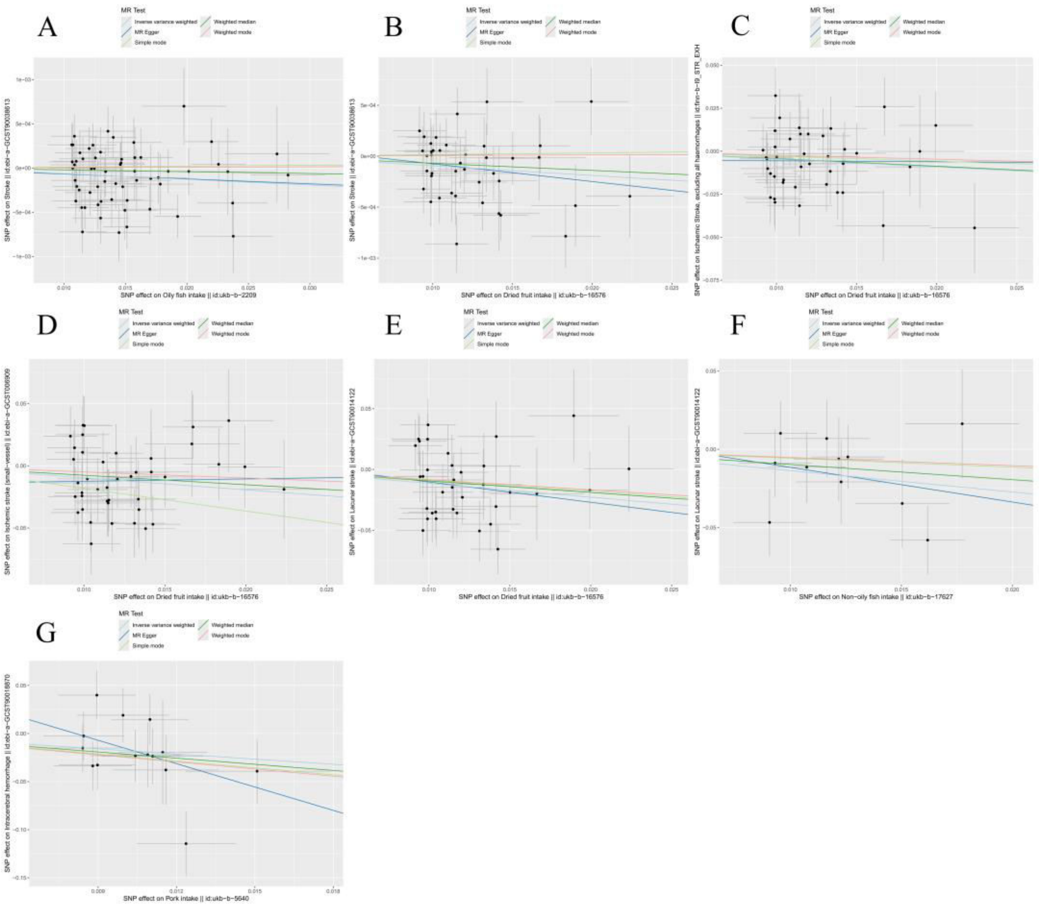

A: Stroke vs Oily Fish Intake,B: Stroke vs Dried Fruit Intake,C: Ischaemic Stroke (Excluding All Haemorrhages) vs Dried Fruit Intake,D: Ischemic Stroke (Small-Vessel) vs Dried Fruit Intake,E: Lacunar Stroke vs Dried Fruit Intake,F: Lacunar Stroke vs Non-Oily Fish Intake,G: Intracerebral Hemorrhage vs Pork Intake.

Fig.S2 Forest Plot of MR Analyses

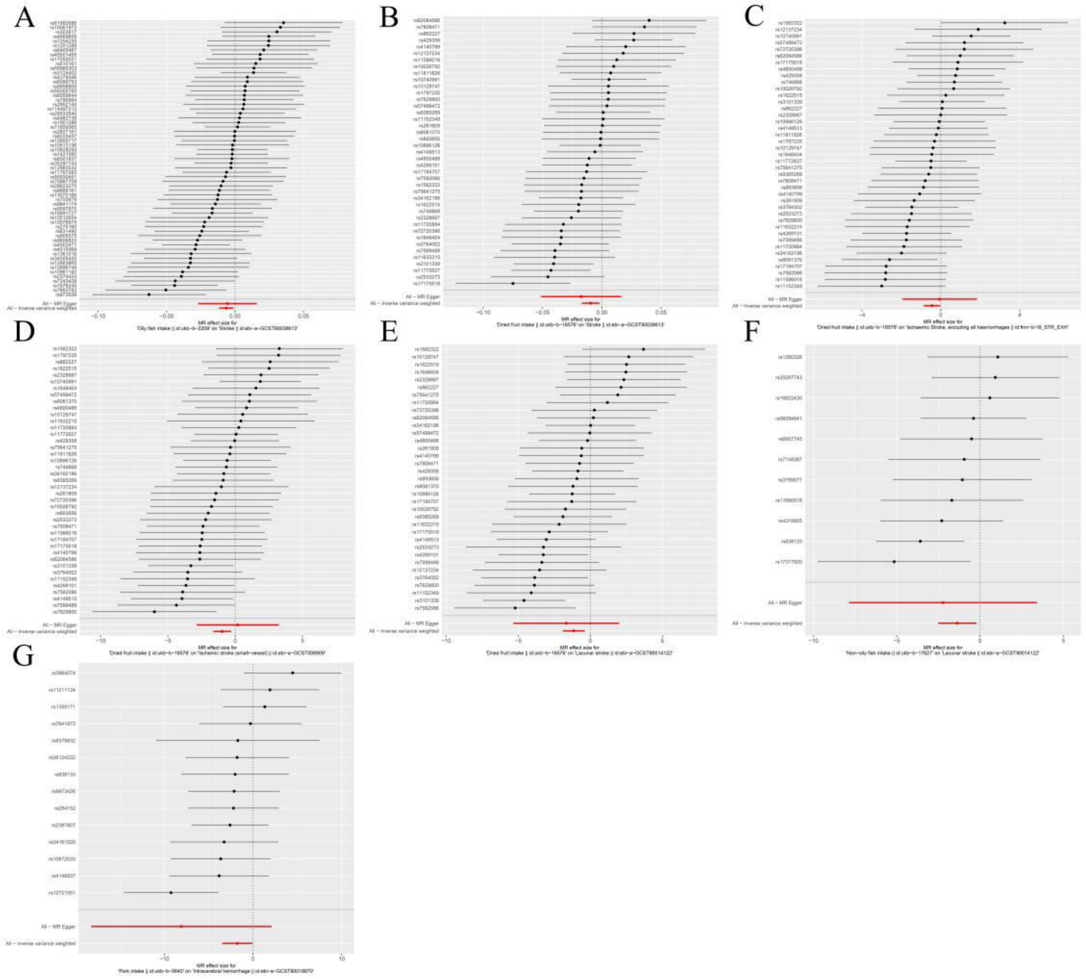

A: Stroke vs Oily Fish Intake,B: Stroke vs Dried Fruit Intake,C: Ischaemic Stroke (Excluding All Haemorrhages) vs Dried Fruit Intake,D: Ischemic Stroke (Small-Vessel) vs Dried Fruit Intake,E: Lacunar Stroke vs Dried Fruit Intake,F: Lacunar Stroke vs Non-Oily Fish Intake,G: Intracerebral Hemorrhage vs Pork Intake.

Fig. S3 Leave-one-out plot of MR Analyses

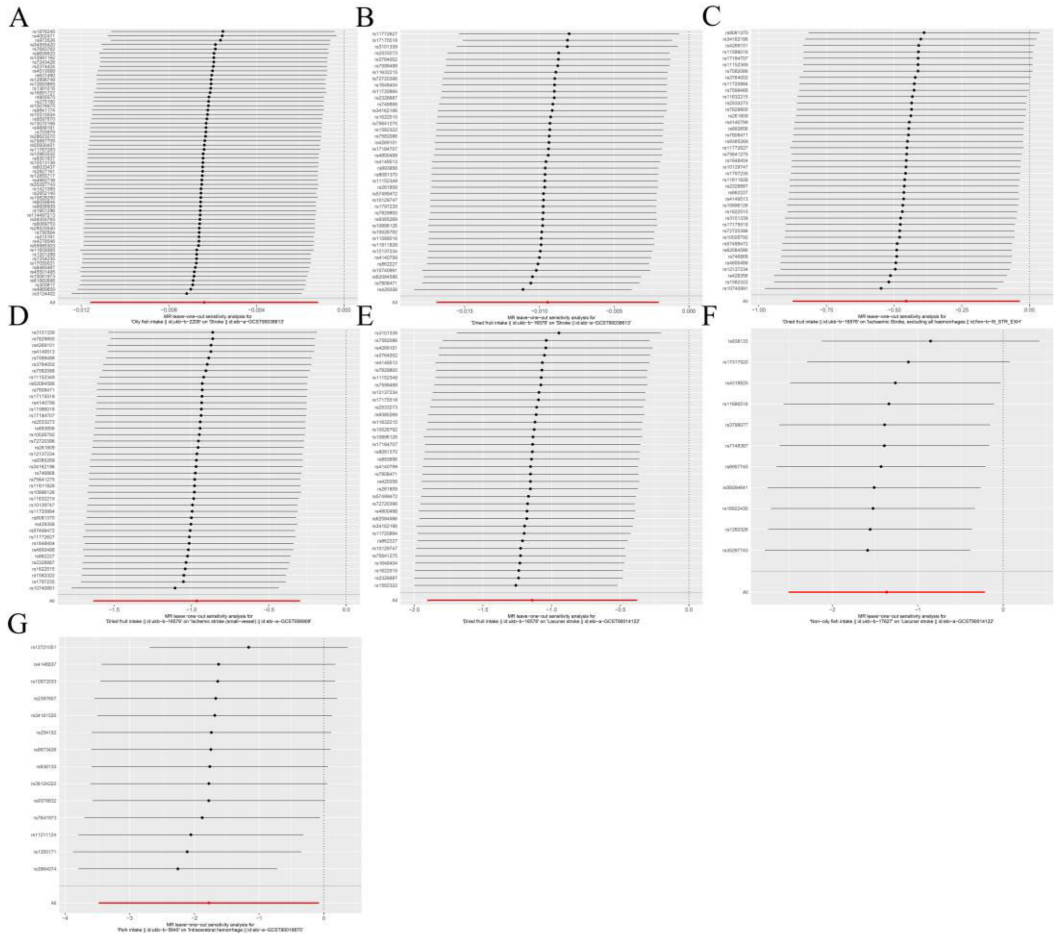

A: Stroke vs Oily Fish Intake,B: Stroke vs Dried Fruit Intake,C: Ischaemic Stroke (Excluding All Haemorrhages) vs Dried Fruit Intake,D: Ischemic Stroke (Small-Vessel) vs Dried Fruit Intake,E: Lacunar Stroke vs Dried Fruit Intake,F: Lacunar Stroke vs Non-Oily Fish Intake,G: Intracerebral Hemorrhage vs Pork Intake.

Fig. S4 Funnel plots of MR Analyses

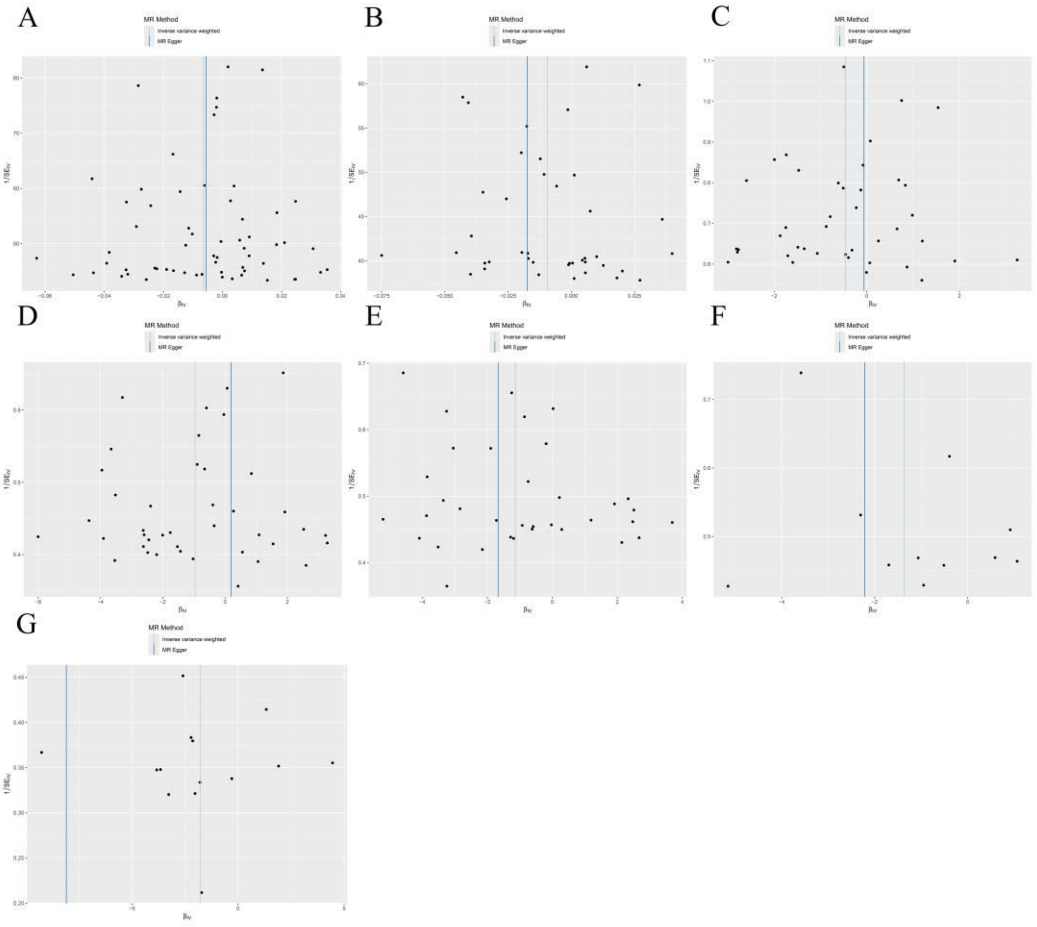

A: Stroke vs Oily Fish Intake,B: Stroke vs Dried Fruit Intake,C: Ischaemic Stroke (Excluding All Haemorrhages) vs Dried Fruit Intake,D: Ischemic Stroke (Small-Vessel) vs Dried Fruit Intake,E: Lacunar Stroke vs Dried Fruit Intake,F: Lacunar Stroke vs Non-Oily Fish Intake,G: Intracerebral Hemorrhage vs Pork Intake.
